# An interdisciplinary model chain quantifies the footprint of global change on reservoir sedimentation

**DOI:** 10.1038/s41598-023-47501-1

**Published:** 2023-11-17

**Authors:** Kilian Mouris, Sebastian Schwindt, María Herminia Pesci, Silke Wieprecht, Stefan Haun

**Affiliations:** 1 Institute for Modelling Hydraulic and Environmental Systems, University of Stuttgart, Stuttgart, Germany; 2 Institute of Hydrology and Water Resources Management, Leibniz University of Hannover, Hannover, Germany

**Keywords:** Hydrology, Hydrology, Geomorphology

## Abstract

Global change alters hydro-climatic conditions, affects land use, and contributes to more frequent droughts and floods. Large artificial reservoirs may effectively alleviate hydro-climatic extremes, but their storage capacities are threatened by sedimentation processes, which in turn are exacerbated by land use change. Envisioning strategies for sustainable reservoir management requires interdisciplinary model chains to emulate key processes driving sedimentation under global change scenarios. Therefore, we introduce a model chain for the long-term prediction of complex three-dimensional (3d) reservoir sedimentation considering concurrent catchment, hydro-climatic, and land-use conditions. Applied to a mountainous Mediterranean catchment, the model chain predicts increased sediment production and decreased discharge for high and medium emission pathways. Increased winter precipitation, accompanied by a transition from snowfall to rainfall, is projected to aggravate reduced summer precipitation, emphasizing a growing need for reservoirs. Additionally, higher winter precipitation proliferates sediment production and reservoir sedimentation. Land use change can outweigh the increased reservoir sedimentation originating from hydro-climatic change, which highlights the significance of localized actions to reduce sediment production. Finally, a 3d hydro-morphodynamic model provides insights into interactions between global change and reservoir sedimentation with spatially explicit information on future sedimentation patterns facilitating the implementation of management strategies.

## Introduction

Global change driven by human legacies since the mid-twentieth century is causing a wide range of hydro-climatic and land use changes that affect the availability of water resources and water distribution^1–5^. Additionally, global warming intensifies impacts on water resources by bolstering evapotranspiration and extreme weather patterns such as more frequent and intense droughts^6^. Large artificial reservoirs for storing water are one of the most powerful tools to buffer the effects of such hydrological extremes. However, reservoir sedimentation threatens buffer capacities by reducing the storage volume and exacerbating local water availability problems^7–10^. Although the loss of storage volume depends on regional characteristics, reservoir sedimentation is a global problem, leading to an annual loss of approximately 0.5–1% in global storage volume^11–15^. This trend has led to a decline in the existing net reservoir storage volume, though more than 3500 new large dams for hydropower production have been built worldwide since 2000^16,17^. Also, the global per-capita storage capacity is shrinking even faster owing to population growth^18^. Moreover, the anticipated hydro-climatic and land use changes are expected to intensify soil erosion and the influx of suspended sediment, hastening the loss of storage volume^19–23^. Predicting reservoir sedimentation and the subsequent storage loss requires precise and holistic assessments of catchment, river, and reservoir processes. Because each system is unique, emulating relevant processes and global change impacts is challenging but necessary for designing reservoirs and implementing targeted reservoir management strategies.

A fundamental challenge is that most of the currently available modeling tools to assess global change impacts lack the necessary level of detail and capacities for simulating the principal processes driving sediment dynamics and reservoir sedimentation. For instance, some models can examine the impact of climate change on the sediment yield and loads for specific catchments^9,19,24–29^ or continents^30,31^, but they neglect the influence of land use change, albeit acknowledging its importance. Other models account for past land use change but do not account for future long-term climate and land use changes^22,32,33^. Only a few existing models are capable of accounting for combined land use and hydro-climatic change impacts on sediment dynamics^34–37^, but they reduce reservoirs to simple lines in one-dimensional hydro-morphodynamic models^38,39^ or use simple empirical estimates^40^ such as the Brune or Churchill curve to assess the effect of climate change on reservoir sedimentation. Such simplistic models have limited relevance for decision-making in reservoir management, which requires explicit knowledge of sediment deposition patterns that a line-like model cannot show. Simplistic reservoir models can still approximate the storage loss of a reservoir, but they cannot account for spatially explicit morphological processes, including bed level changes such as deposition delta evolution. Furthermore, simplified models do not consider recirculation zones, lateral inflows, the influence of the outflows (e.g., turbine operation), and other complex 3d hydrodynamics. However, such information is essential for the development and implementation of appropriate and sustainable reservoir management strategies. For example, venting of rapid sediment-laden flows on the reservoir bottom, referred to as turbidity currents^41^, and other sediment routing actions (e.g., sluicing) require a deep understanding of 3d hydrodynamics. Also, sediment deposits in front of the bottom outlets of a dam pose a significant risk to the safe operation of a reservoir^42^, which can be alleviated through local dredging or flushing operations. Both dredging and spatio-temporally efficient flushing require 3d information on hydro-morphodynamic processes, but currently, no modeling system or chain provides such information. Thus, state-of-the-art modeling tools do not imply multidisciplinary simulations needed to predict reservoir sedimentation processes and patterns in the long term and in light of global change scenarios.

To address these challenges, we present a novel model chain that uses information on catchment physics, including the hydro-climatic state and land use to predict long-term sediment dynamics and multi-dimensional reservoir sedimentation processes. The process-based model chain accounts for changes in temperature, precipitation, discharge, sediment yield, and reservoir sedimentation, by also considering the geometry and operating scheme of the reservoir. The centerpiece of the model chain is a three-dimensional (3d) numerical model, which predicts flow dynamics and sediment transport and enables us to show how different global change scenarios impinge on reservoir sedimentation processes.

## Methods

### Model chain and application example

**Figure 1:**
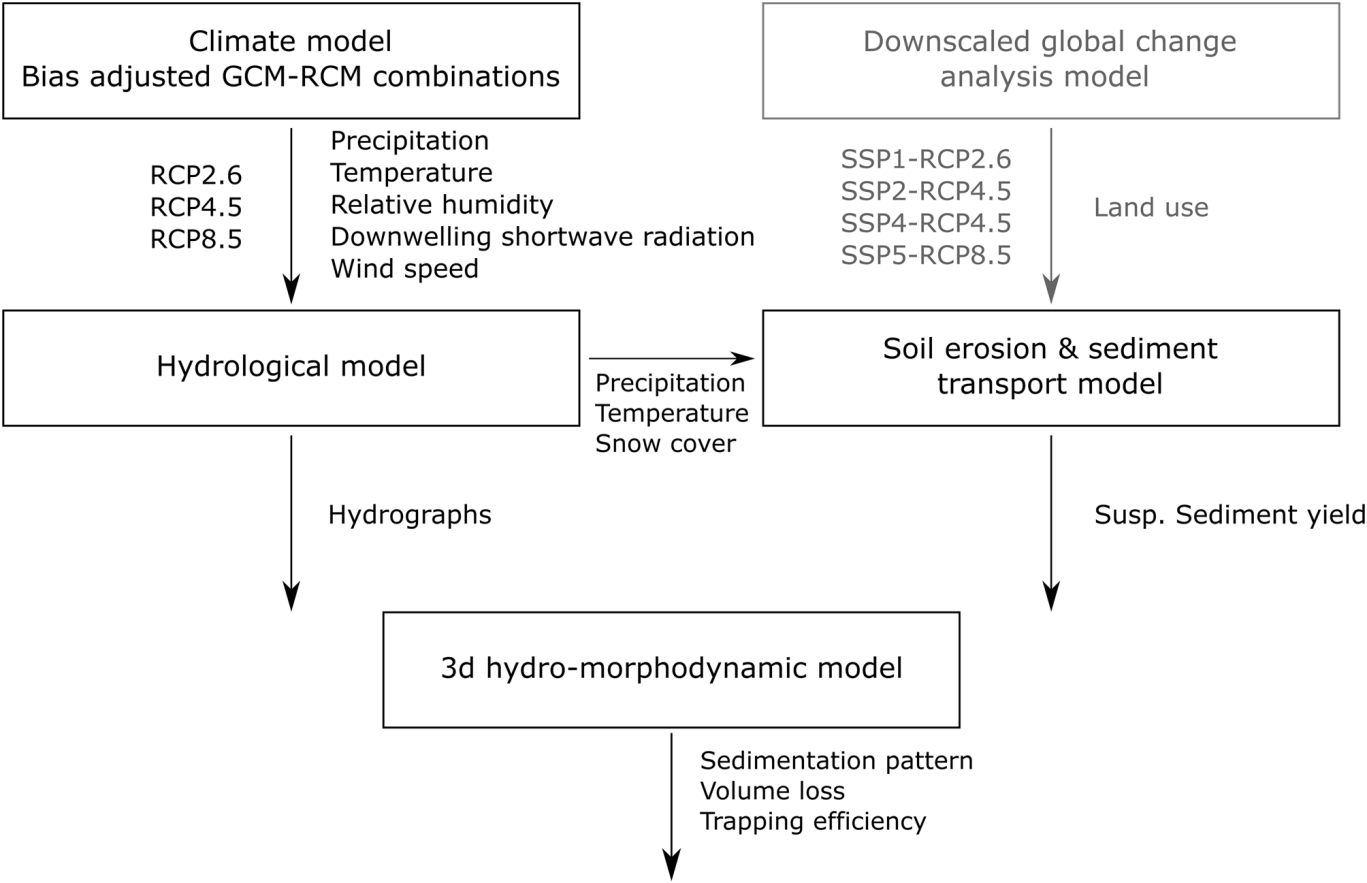
Model chain simulating the effects of global change on reservoir sedimentation as a function of land use (in gray and by means of Shared Socioeconomic Pathways, SSPs) and climate change based on Representative Concentration Pathways (RCPs), and three Global Climate Models (GCMs) with local refinement through two Regional Climate Models (RCMs).

The process-based model chain assesses the effect of climatic, land use, and resulting hydrological changes on reservoir sedimentation. First, the primary impacts of climate change are predicted for three Representative Concentration Pathways (RCPs) using three different climate models, including near-surface temperature and precipitation. To predict secondary climate change impacts resulting from temperature and precipitation changes, a state-of-the-art hydrological model, a soil erosion plus sediment transport model, and a 3d hydro-morphodynamic reservoir model are set up and combined, to benefit from their specialization and the possibility to correctly account for physical processes at different scales. In addition, datasets derived from a downscaled global change analysis model^43^ enable the emulation of future land use change for four Shared Socioeconomic Pathways (SSP-RCPs). The model chain served to simulate future projections of sediment trapping in a reservoir as a function of three scenarios of hydro-climatic change and four scenarios of combined climate and land use change (Fig. 1).

Although individual model input parameters are calibrated, the output is still subject to uncertainty that propagates through the entire model chain and leads to superposition effects. Additional uncertainty stems from long-term predictions of climate projections, which exceed the inherent uncertainty of the calibrated input parameters^44^. Finally, the model chain enables long-term process simulation to examine the influence of climate and land use changes on reservoir sedimentation, where quantitative outputs are still subject to uncertainty.

**Figure 2:**
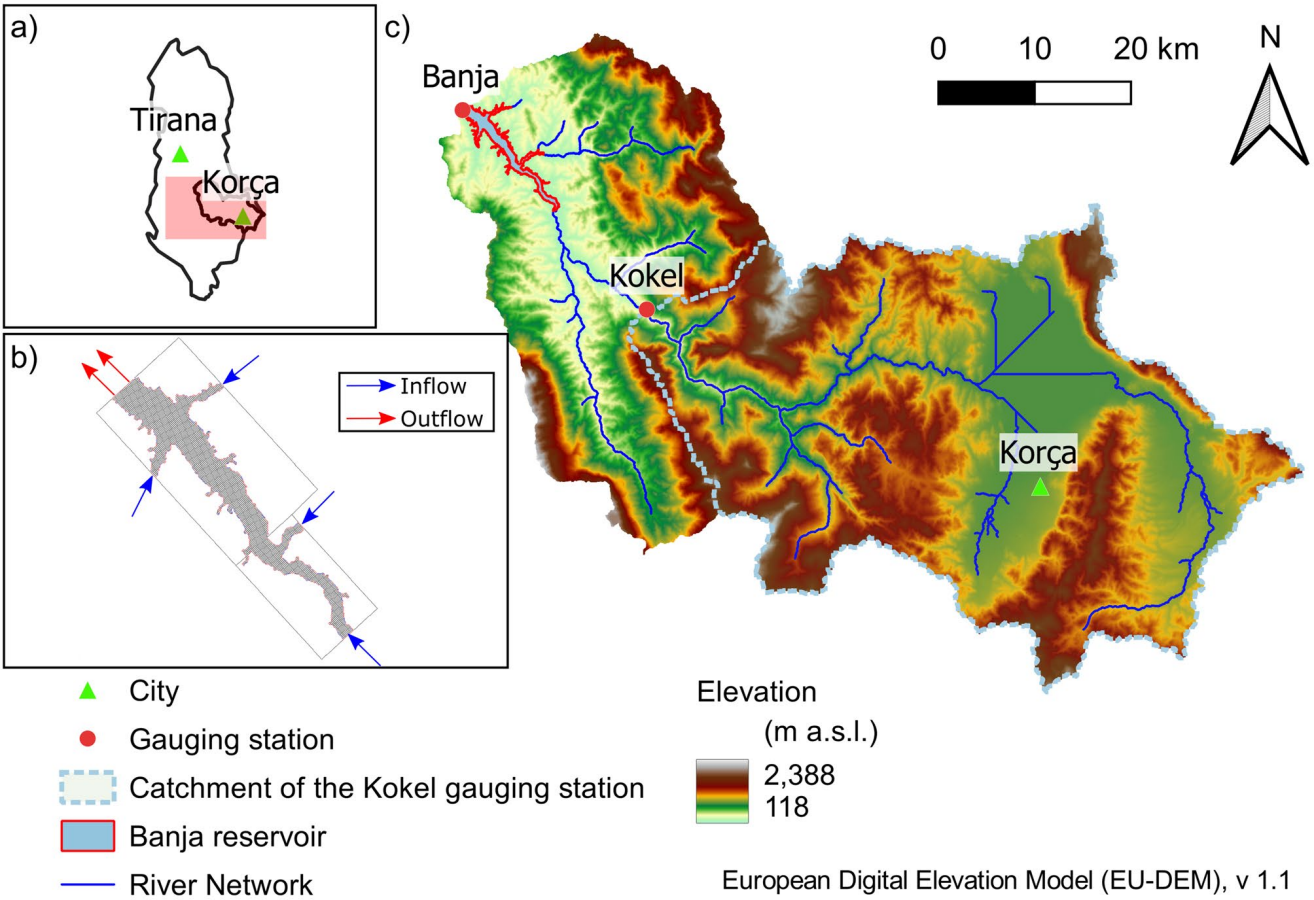
Location of the Mediterranean showcase catchment (**a**) in Albania, (**b**) the extent of the 3d hydro-morphodynamic numerical model including tributaries (blue arrow) and outflows (red arrows), and (**c**) the catchment topography with gauging stations, sub-catchments, and location of the Banja reservoir. The figure was created by the authors using QGIS3.18.1 (https://qgis.org/en/site/).

An application of the model chain showcases the Banja reservoir in the Devoll catchment in Southeastern Albania (Fig. 2) with a typical Mediterranean climate featuring high erosion rates and vulnerability to climate change^45–47^. The emerging region is experiencing major land use changes and large investments in hydropower^48^. The mountainous Devoll catchment spans 2900 km^2^ with elevations ranging from 113 to 2390 m a.s.l. The land use is currently characterized by forest (30%), scrub and herbaceous vegetation (25%), and agriculture (25%). Over recent decades, land use has undergone substantial changes, particularly after the collapse of communism, and is increasingly influenced by global market principles^49^. Dry hot summers and wet winters characterize the Mediterranean hydro-climate with low summer and high winter and spring flows. In winter, high elevations of the catchment are frequently covered by snow leading to a precipitation and snowmelt-driven flow regime. High rainfall erosivity on steep slopes with poorly aggregated soils contributes to high sediment production and sediment yields^50,51^ leading to a great potential for reservoir sedimentation of existing and planned reservoirs. Commissioned in 2016, the Banja reservoir has a length of 14 km, a maximum water depth of 60 m near the dam, a surface of 14 km2, and a maximum storage capacity of 400 million m^3^. A further upstream-located reservoir, commissioned in 2020, was not included in this study because ground truth data were not available at the time of calibration.

### Available ground truth data

Ground truth data on discharge and suspended sediment concentrations (SSCs) were obtained from the Kokel gauging station (Fig. 2) for a period between May 2016 and April 2018, when the water depth exceeded a minimum measurement threshold of 1 m. Discharge and SSC were monitored with two stationary-mounted horizontal acoustic Doppler current profilers (H-ADCPs)^52^.

A digital elevation model (DEM) of the bathymetry was generated based on a drone survey prior to the reservoir filling in 2016. In 2019, the bathymetry of the reservoir was re-assessed using moving ADCP measurements. The 2016 and 2019 topography recordings were projected on a numerical mesh and served to calculate the height of sediment deposits in the reservoir. Calibration of the 3d numerical model was performed based on bed level changes along the thalweg. During a field survey in 2021, sediment samples were collected from the reservoir bed at 27 locations in the reservoir using an Ekman grab sampler that samples the uppermost 20 cm of the deposits. Sampling was carried out in both deep (> 40 m) and shallow (near tributaries) areas of the entire reservoir. The grain size distributions of the samples were determined with a portable particle size analyzer based on laser diffraction and revealed that the sediment depositions predominantly consisted of fine sediments with cohesive characteristics (< 63 µm). A laboratory analysis of the deposited sediments showed dry bulk densities ranging from 726 to 950 kg m^−3^.

### Climate and land use projections

The impacts of climate change on reservoir sedimentation are estimated for three RCPs using ensembles of three Global Climate Models (GCMs), which are dynamically downscaled by two different Regional Climate Models (RCMs). To mitigate potential bias stemming from the selection of climate models, specific model combinations with similar climate trends for precipitation and temperature were grouped. One climate model from each group was used to represent the large variety of GCMs while using only 3 GCM-RCM combinations (Supplementary Information, Table S1). The selected combinations (Supplementary Information, Table S1) are considered representative of the large variety of GCM-RCMs. The climate models provide meteorological information on total precipitation, near-surface temperature, near-surface relative humidity, surface downwelling, shortwave radiation, and near-surface wind speed using the MultI-scale bias AdjuStment (MidAS v0.2.1) tool^53^ for correcting daily mean values and the ERA5 reanalysis dataset^54^ as reference data. The resulting projections have a spatial resolution of 0.11 degrees and a temporal resolution of 3 h over a period from 01/1981 to 12/2100.

The here-used three RCPs encompassed a low greenhouse gas emissions pathway (RCP2.6), a medium greenhouse gas emissions pathway (RCP4.5), and a high greenhouse gas emissions pathway (RCP8.5). In combination with hydro-climatic change scenarios, land use projections^43^ were implemented in the model chain (Fig. 1) through four Shared Socioeconomic Pathways (SSPs). The SSPs embrace greenhouse gas emissions and account for climate change, population growth, economic development, and technological advancement, thereby offering more holistic global change scenarios^55^ in accordance with the Coupled Model Intercomparison Project Phase 6 (CMIP6) design^56^. Thus, catchment responses to sustainable development (SSP1-RCP2.6), middle-of-the-road development (SSP2-RCP4.5), unequal development (SSP4-RCP4.5), and fossil-fueled development (SSP5-RCP8.5) were evaluated in combination with the GCMs, RCMs (Table 1; more detail in Supplementary Information, SI1). In total, the model chain was run for 21 scenarios comprising 3 RCP and 4 SSP-RCP scenarios, each using 3 GCM-RCM combinations. The mean values of the climate model ensemble served to obtain robust trends and derive a range of possible outcomes due to the spread of climate projections (see details in Pesci et al.^44^).

**Table 1:** Investigated scenarios to analyze climate change (RCP) and global change (SSP-RCP) impacts on reservoir sedimentation.

RCP (climate change only)	SSP-RCP (global change)
RCP2.6 (low emissions)	SSP1-RCP2.6 (sustainability)
RCP4.5 (stabilized emissions)	SSP2-RCP4.5 (middle of the road)
	SSP4-RCP4.5 (inequality)
RCP8.5 (high emissions)	SSP5-RCP8.5 (fossil-fueled development)

**Figure 3:**
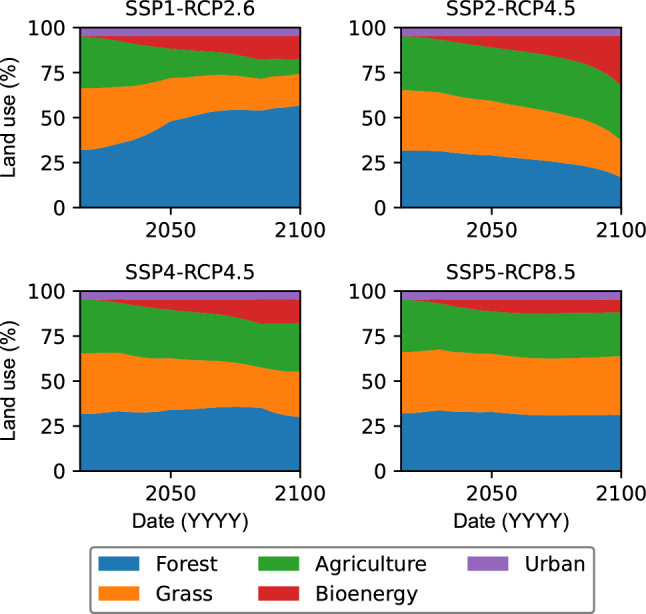
Projected land use changes in the Devoll catchment for the four investigated global change scenarios (combinations of RCPs and SSPs).

Figure 3 illustrates the four global change scenarios for the Devoll catchment, indicating the major land use classes and their projected changes by 2100. The urban land use category shows almost no change and constitutes approximately 5% of the total area. In contrast, the distribution of crops, forests, and grasslands varies considerably across the scenarios. In the SSP1-RCP2.6, both forest and grassland areas are projected to experience a substantial increase of up to 70% by 2100. Conversely, the SSP2-RCP4.5 and SSP4-RCP4.5 scenarios predict a decline in forest and grassland cover by 40% and 55%, respectively. In both RCP4.5 scenarios, the decline in forest and grassland in the far future is primarily caused by the expansion of agricultural land, particularly for cultivating bioenergy crops. For example, the SSP2-RCP4.5 scenario shows a significant increase in energy crop production due to the requirement of reducing greenhouse gas emissions with socioeconomic trends following their original course. Bioenergy crops are commonly used grain-based crops, such as rapeseed, corn, and sunflower. In the SSP5-RCP8.5 scenario, the land use changes marginally, as technological progress is achieved through fossil-fueled development. Popp et al.^57^ provide more details on land use projections for various SSPs.

### Hydrological model

The hydrological processes in the catchment are implemented in the model chain in the form of the Water Flow and Balance Simulation Model (WaSiM^58,59^) using the process-oriented Richards approach, with an additional sub-model for snow interception under forest canopies^60^. The model domain is defined at a spatial resolution of 1 km^2^ and a temporal resolution of 3 h. In the case of the Devoll catchment, the calibration period spanned from May 2016 to April 2018 for which discharge measurements were available. WaSiM was initiated with a warm-up period of one year (May 2015 to April 2016). WaSiM produces hydrographs that constitute the liquid upstream boundary for the 3d hydro-morphodynamic model. Snow cover was estimated by WaSiM based on an energy balance approach and served as input for the soil erosion and sediment transport model. Missing information on the climate variables of relative humidity, wind speed, and global radiation was interpolated through inverse distance weighting. In addition, missing precipitation and temperature data were derived with a combination of elevation-dependent regression and inverse distance weighting in the model. More detailed information on the hydrological model, its calibration and validation, and the selected modeling approaches is provided in Supplementary Information SI2.

### Soil erosion and sediment transport model

In the model chain, the Revised Universal Soil Loss Equation (RUSLE)^61^ serves to predict gross soil erosion, and the SEdiment Delivery Distributed (SEDD)^62^ model estimates the sediment delivery and transport at the catchment scale. The predicted monthly suspended sediment yields constitute the solid-materials upstream boundary of the 3d hydro-morphodynamic reservoir model. A semi-automated (Python) workflow evaluates the combination of the RUSLE and SEDD model to account for the non-erosivity of snowfall and the erosivity of snowmelt by introducing a seasonal memory into the RUSLE. In the case of the Devoll catchment, the combined soil erosion and sediment transport model was calibrated using suspended sediment load measurements from 05/2016 to 04/2018^50^. Alternative methods for estimating sediment concentrations, such as constant concentration-discharge relationships, are not suitable because they are likely to vary with climate change. The key advantage of choosing the RUSLE-SEDD combination is the efficient consideration of future changes in land use, precipitation, and temperature (Fig. 1). To implement the calculated Suspended Sediment Yield (SSY) into boundary conditions for the next model element, it must be converted into a time-discrete Suspended Sediment Concentration (SSC). SSY is the total suspended sediment transported by the river (or through the outlet of a catchment) over a specific period, and SSC refers to the concentration of sediment particles suspended in the water column. Hence, the SSC is calculated back from the monthly SSY and is therefore constant for each month. Detailed information on the RUSLE-SEDD, the validation, and the conversion of SSY to SEDD are provided with the Supplementary Information SI3.

### 3d hydro-morphodynamic model

The centerpiece of the interdisciplinary model chain for the coupled simulation of hydro-morphodynamic processes driving reservoir sedimentation is a 3d numerical model (SSIIM 2-Sediment Simulation In Intakes with Multiblock Option)^63^. 3d modeling is particularly important to represent variations in vertical suspended sediment concentrations, velocities, and the complex three-dimensional flow field with helical flows. For modeling reservoir sedimentation resulting from fine sediment deposition, multiple grain sizes are considered in the model. For the Banja reservoir, the model accounted for four inflow and two outflow boundaries (spillway and turbine inlet), with inflow discharges and inflowing sediment derived from the hydrological model and the soil erosion and sediment transport model, respectively. The outflow was calculated as a function of the reservoir water level, the inflow, the storage curve, and site-specific operating rules that target a seasonal water level. The model calibration was performed based on the observed bed changes between the bathymetric surveys conducted in 2016 and 2019.

Since the computing time of a hydro-morphodynamic numerical 3d model tends to take several weeks to months (Supplementary Information, SI4), several simplifications were made to obtain acceptable runtimes for predicting global change impacts by 2100. For example, the resolution of the computational mesh may be as coarse as 50-m edge lengths, which then require specific turbulence models, such as the Reynolds-averaged Navier–Stokes (RANS) equations. Furthermore, SSIIM2 uses an implicit solver for the Navier–Stokes equations, which allows the use of large time steps (5400 s) and consequently reasonable computing times^64,65^. Also, additional algorithms, such as flow limiters, were implemented for computational stability in flat or triangular cells near the reservoir banks that may result from the wetting and drying algorithm. The advantage is that only wetted cells are considered in SSIIM2, which reduces the number of cells during calculation, especially when the water level changes, but also when reservoir sedimentation occurs.

The physical simplifications and numerical workarounds are expressed in the numerical model by calibration parameters that must be adjusted individually for each reservoir^66^. In the context of calibration, the evaluation of uncertainties is of paramount importance and can only be estimated by data-driven approaches, such as Bayesian calibration^67,68^. Still, 3d-modeling is physically more precise than often-used 1d or 2d hypotheses that reduce the complex flow patterns in a reservoir to a geometric line or plan. As a result of detailed spatial modeling of hydro-morphodynamic processes, the related uncertainties are lower^69^, leading to less risk of equifinality^70^. Further details on the 3d hydro-morphodynamic model and the developed codes used to generate the upstream and downstream boundary conditions can be found in Supplementary Information SI4.

## Results

The process-based model chain was used to assess the impact of hydro-climatic and land use changes on reservoir sedimentation and its preceding processes in the Devoll catchment and Banja reservoir from January 1981 to December 2100. The first 30 years (1981–2010) served as a reference period for comparison with three future periods: the 2011–2040 period represents the near future, the 2041–2070 period the mid future, and the 2071–2100 period the far future. The subsequent reservoir sedimentation of the Banja reservoir was simulated from impoundment in 2016 to December 2100 (84 years).

### Climate change impacts

#### Temperature and precipitation

**Figure 4:**
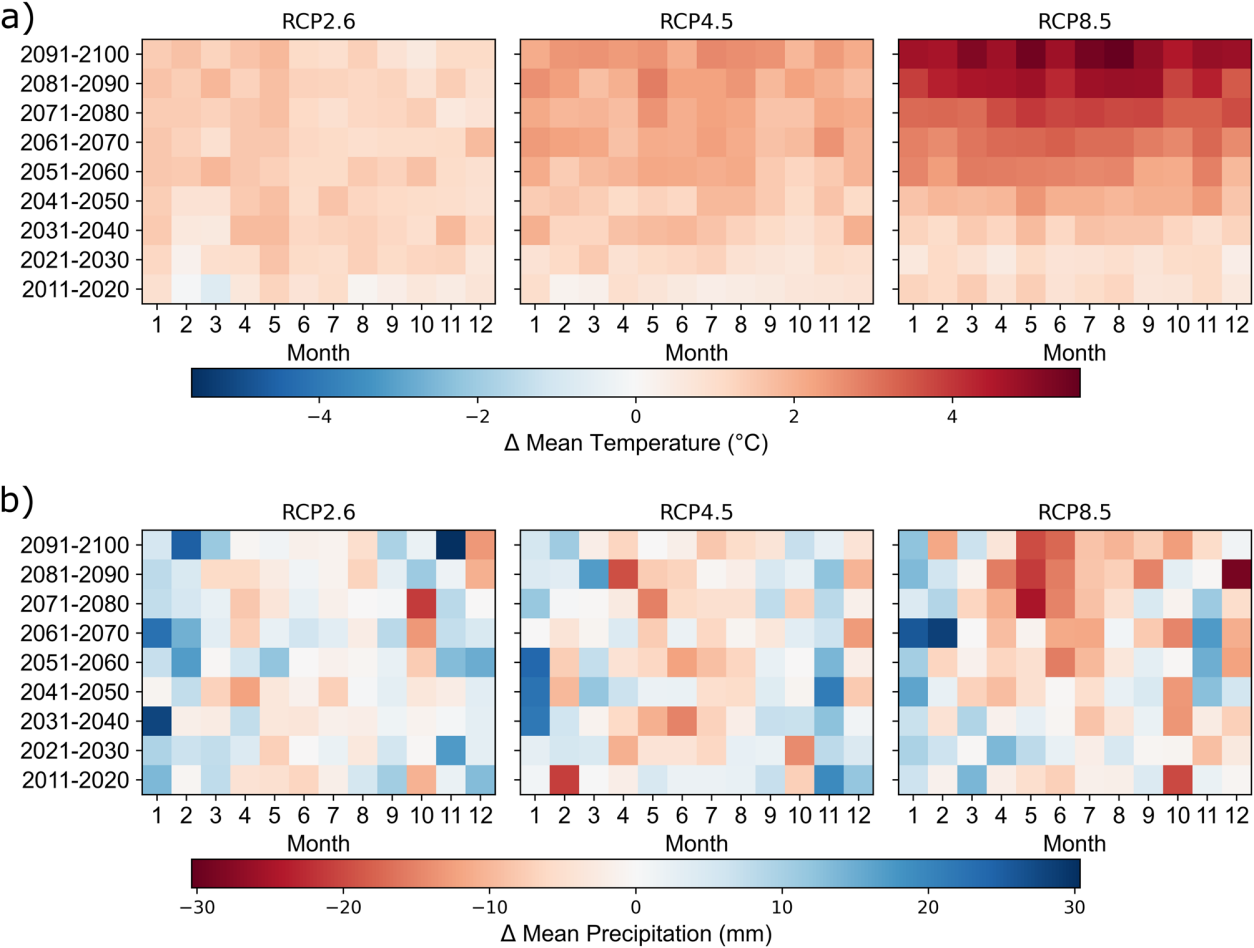
Decadal changes in monthly and spatial averages for the Mediterranean Devoll catchment relative to the reference period (1981–2010). (**a**) Average monthly temperature change (°C) and (**b**) average monthly precipitation change (mm). The figure was created by the authors using Matplotlib 3.5.1 (https://matplotlib.org/).

Climate change primarily affects temperature and precipitation (primary climate change impacts) which drive hydrological and sediment-related processes, such as discharge, soil erosion, and the transport of sediments into the reservoir (secondary climate change impacts). The mean annual temperature in the catchment increases the most for RCP8.5, notably by 2.5 °C for the mid future and by 4.3 °C for the far future compared to the reference period (Fig. 4a and Supplementary Information, Fig. S4). The temperature increase is smaller for medium (RCP4.5) and low (RCP2.6) emissions for the mid (1.8 °C and 1.3 °C, respectively) and (2.2 °C and 1.3 °C, respectively) far future. Also, the seasonal temperature trends are expected to remain nearly unchanged for all RCPs, with slightly higher temperature increases in summer compared to winter, particularly for RCP8.5 (Fig. 4a).

Predicted changes in precipitation patterns are less clear (Fig. 4b), with trends toward more winter (January to March) and less summer precipitation. Thus, typical Mediterranean precipitation patterns of wet winters and dry summers can be expected to slightly intensify (Supplementary Information, Fig. S5). This trend is evident across all emission RCPs but is most pronounced for RCP8.5. In the far future, total annual precipitation is expected to increase by 2–4% for RCP2.6, while total annual precipitation is expected to decrease by up to − 9% for RCP8.5 (Supplementary Information, Table S7). No considerable total precipitation changes are expected for RCP4.5

#### Discharge and suspended sediment yield

As a result of higher temperatures (Fig. 4a), mean annual snow storage is projected to decrease substantially in all RCPs, with the largest decrease of 83% anticipated for RCP8.5 and the smallest decrease of 36% for RCP2.6 in the far future (2071–2100) (Supplementary Information, Table S7, and Fig. S6).

**Figure 5:**
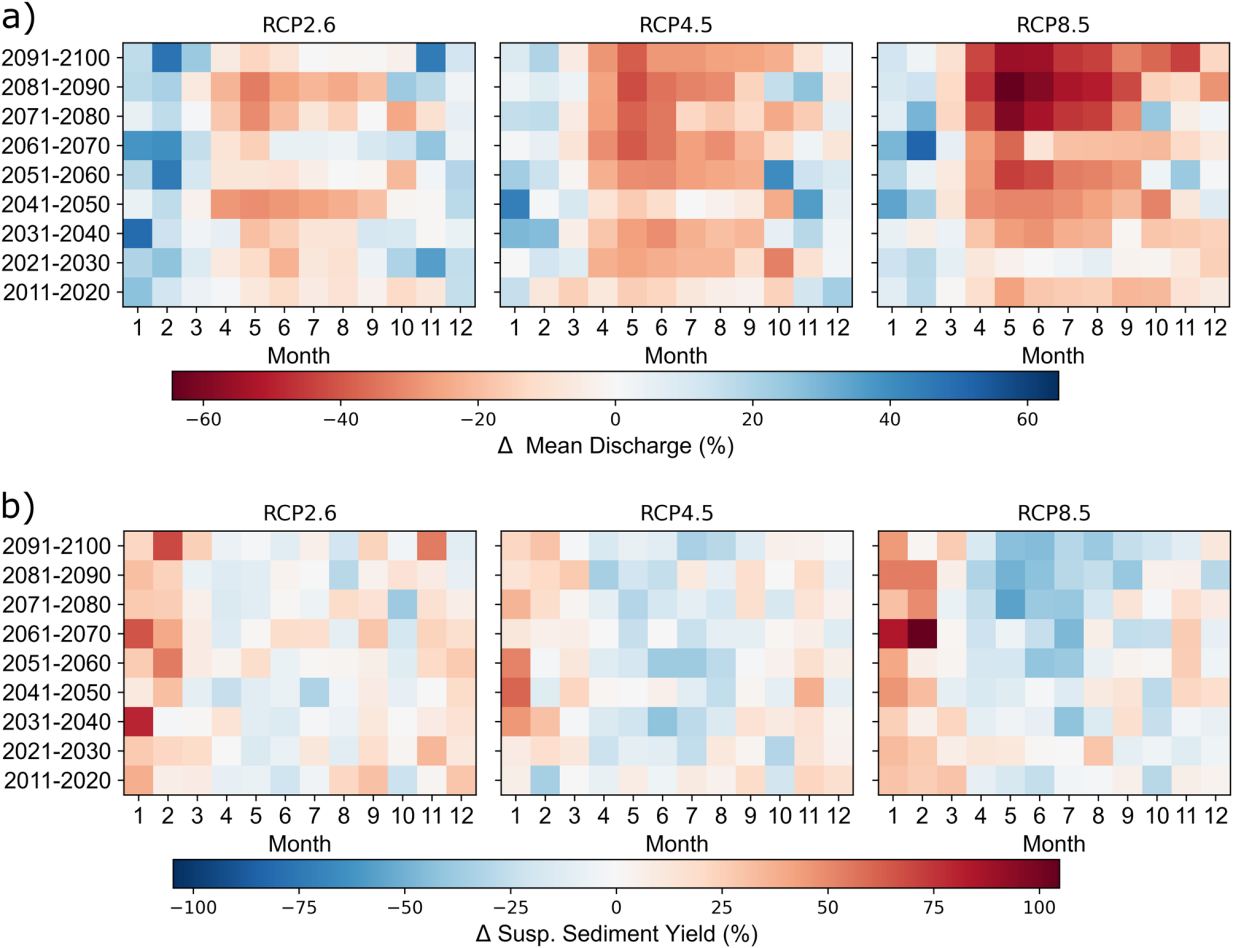
Decadal percent changes relative to the reference period (1981–2010) for the three climate change scenarios investigated. (**a**) Changes in mean monthly discharge (%) and (**b**) changes in mean monthly suspended sediment yield (%) of the Mediterranean Devoll River. The figure was created by the authors using Matplotlib 3.5.1 (https://matplotlib.org/).

Similar to precipitation, RCP2.6 results in a higher mean annual discharge than the other scenarios, most prominently in the near to mid future with an increase of up to 6%. However, the mean annual discharges show a declining trend in both the RCP4.5 and RCP8.5, with an accelerated decrease over time. Particularly for RCP8.5, the mean annual discharge is expected to decrease by more than 20% in the far future (Supplementary Information, Table S8). The seasonal variations are similar for the three RCPs and intensify with increasing emissions, with spring and summer discharge decreasing (e.g., by more than 40% in May) and January and February discharge increasing (Fig. 5a). Due to the changes in the precipitation regime, the decrease in snow storage, and the earlier snowmelt, the discharge peak is predicted to shift from April to March (Supplementary Information, Fig. S7). While annual and monthly discharge averages exhibit decreasing trends, the occurrence of extreme events such as floods with a 50-year return period increase by 7% (RCP2.6), 11% (RCP4.5), and 19% (RCP8.5) in the far future, also effecting on soil erosion.

The annual suspended sediment yield (SSY) of the Mediterranean Devoll catchment is expected to increase from 1.2 million tons year^−1^ by up to 9% for RCP2.6 and by up to 4% for RCP4.5 (Supplementary Information, Table S8). Despite a decrease in precipitation and discharge for RCP8.5, the simulations show an increase in SSY by 5% in the near to mid future. Only in the far future will the SSY also decrease by approximately 3%. The predicted seasonal changes are similar to the predictions for precipitation and discharge and show a considerable increase in the winter months and a decrease in spring and summer (Fig. 5b). In contrast to the annual SSY, the mean annual SSC increases for all emission scenarios but most substantially for RCP8.5 (27%) in the far future (Supplementary Information, Table S8). The increase in SSC for RCP2.6 is the lowest and ranges from 3 to 8%. The resulting predictions of discharge and sediment yield control the amounts of water and sediment arriving at the upstream boundary of the 3d reservoir sedimentation model.

#### Reservoir sedimentation

**Figure 6:**
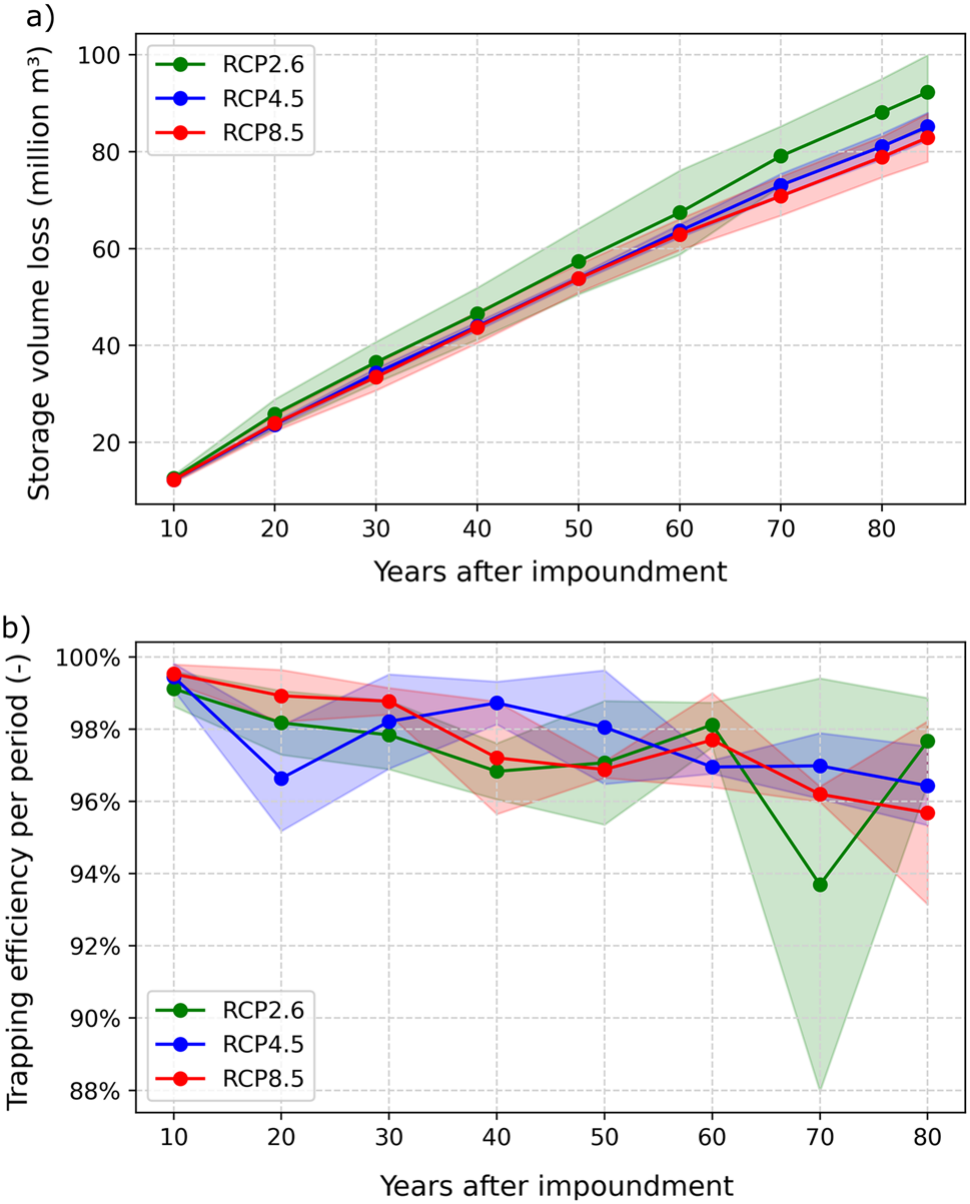
Evolution of (**a**) the loss in storage volume after impoundment and (**b**) the trapping efficiency at 10-year periods for the Banja reservoir and the three investigated RCPs. The shadowed areas represent the spread of ensemble climate projections, calculated as the mean value ± standard deviation.

The predicted loss in storage volume of the Banja reservoir was most prominent for RCP2.6 (Fig. 6a). Specifically, the loss is estimated to be 23% after 85 years since the impoundment and is caused by the highest sediment flow along with the highest discharges for RCP2.6. However, the uncertainty in the climate projections is highest for RCP2.6 which is indicated by the high standard deviation (Fig. 6a). The storage volume loss for RCP4.5 and RCP8.5 is similar with approximately 21%. Interestingly, in the far future, the storage volume loss for RCP4.5 slightly surpasses that of RCP8.5, which can be explained by the declining SSY associated with RCP8.5 in the far future. In addition, the spread of climate projections is smallest for RCP4.5, where all climate projections within the ensemble resulted in similar volume losses.

The sedimentation rate and subsequent decrease in reservoir storage volume are not only determined by the sediment inflow but also by the trapping efficiency (TE, Fig. 6b), which depends on geometric reservoir characteristics and its operation. TE represents the ratio of the deposited sediments to the time-integrated sediment inflow over a certain period. During the first simulation decade, nearly all inflowing sediment is trapped, resulting in a TE exceeding 99%. TE decreases for all RCPs to 95.8 to 97.2% after 80 years of impoundment as a result of changed hydrodynamics because of bathymetric change (i.e., fine sediment deposition). Yet, the trend is not generally continuous. For instance, in the case of RCP2.6, TE exhibits an initial increase to more than 98% after 50–60 years but decreases abruptly afterward. This fluctuation is caused by a predicted wet season with exceptionally high inflows over several weeks in one of the climate projections within the RCP2.6 ensemble, leading to a decrease in TE and also contributing to increased uncertainty. The TE trends are subjected to significant uncertainty due to the variability in climate projections and the resulting timing of flood events, which means that a statistically significant difference between the RCPs cannot be identified.

**Figure 7:**
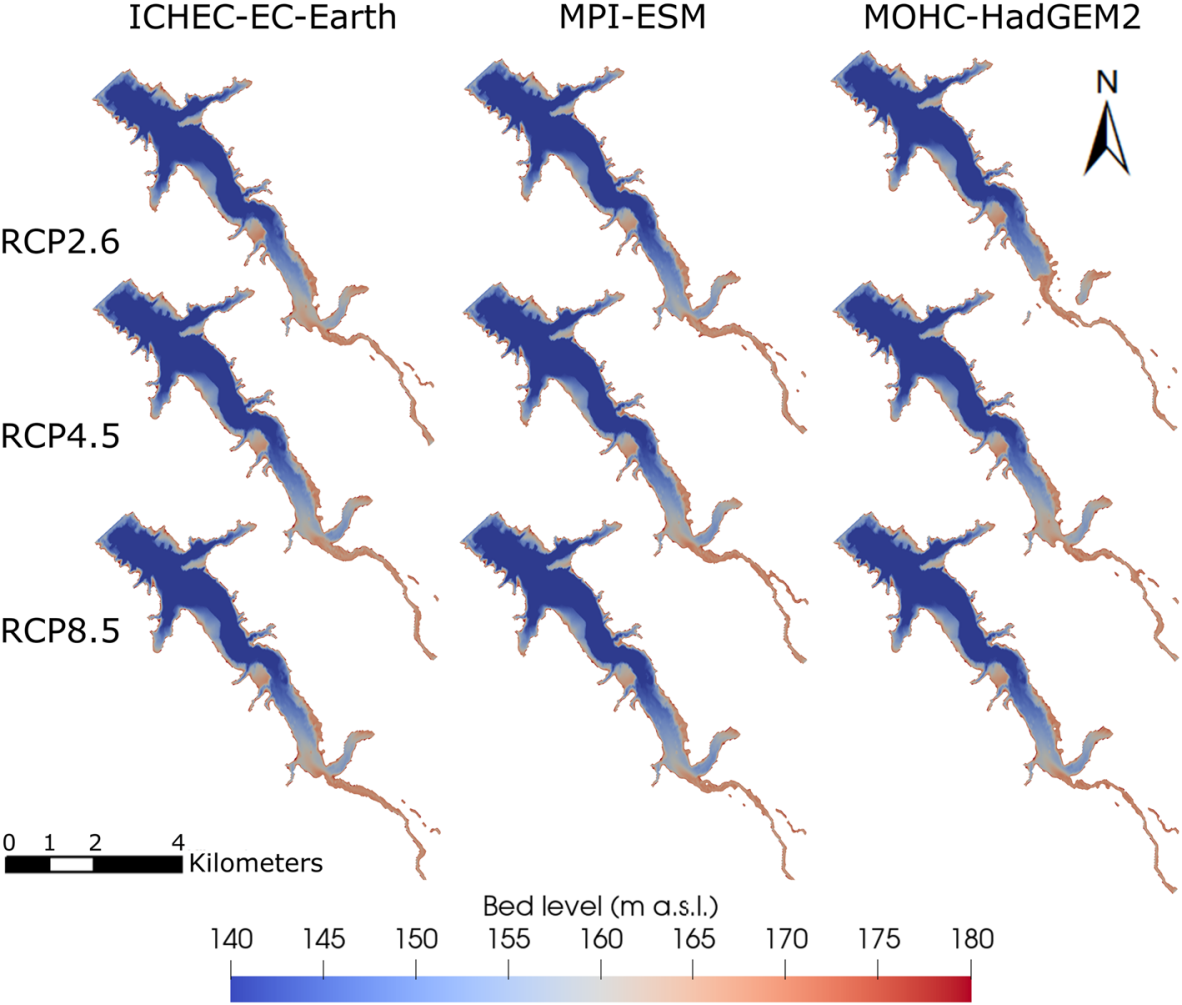
Comparison of reservoir bed levels across three RCPs and three climate models after 84 years of operation (2100).

The reservoir bed levels after 84 years of operation show similar sedimentation patterns across the RCPs and climate models (Fig. 7). Following the commissioning of the dam, the reservoir is in a deposition regime, characterized by ongoing sedimentation primarily concentrated at the head of the reservoir. As sedimentation progresses, the deposition delta gradually moves in the downstream direction. Consequently, a river channel develops in the upstream section of the reservoir, which reaches a state of sediment balance (see bed level evolution in Supplementary Information, SI9). The channel and delta progression extends slightly further downstream in RCP2.6 because the sediment yield is higher compared to RCP4.5 and RCP8.5 (Fig. 7). In addition, the substantial sediment deposition at the confluence of the two main tributaries causes the eastern tributary in RCP2.6 to be temporally disconnected for MOHC-HadGem2 at low water levels.

### Global change impacts through combined and isolated climate and land use change

**Figure 8:**
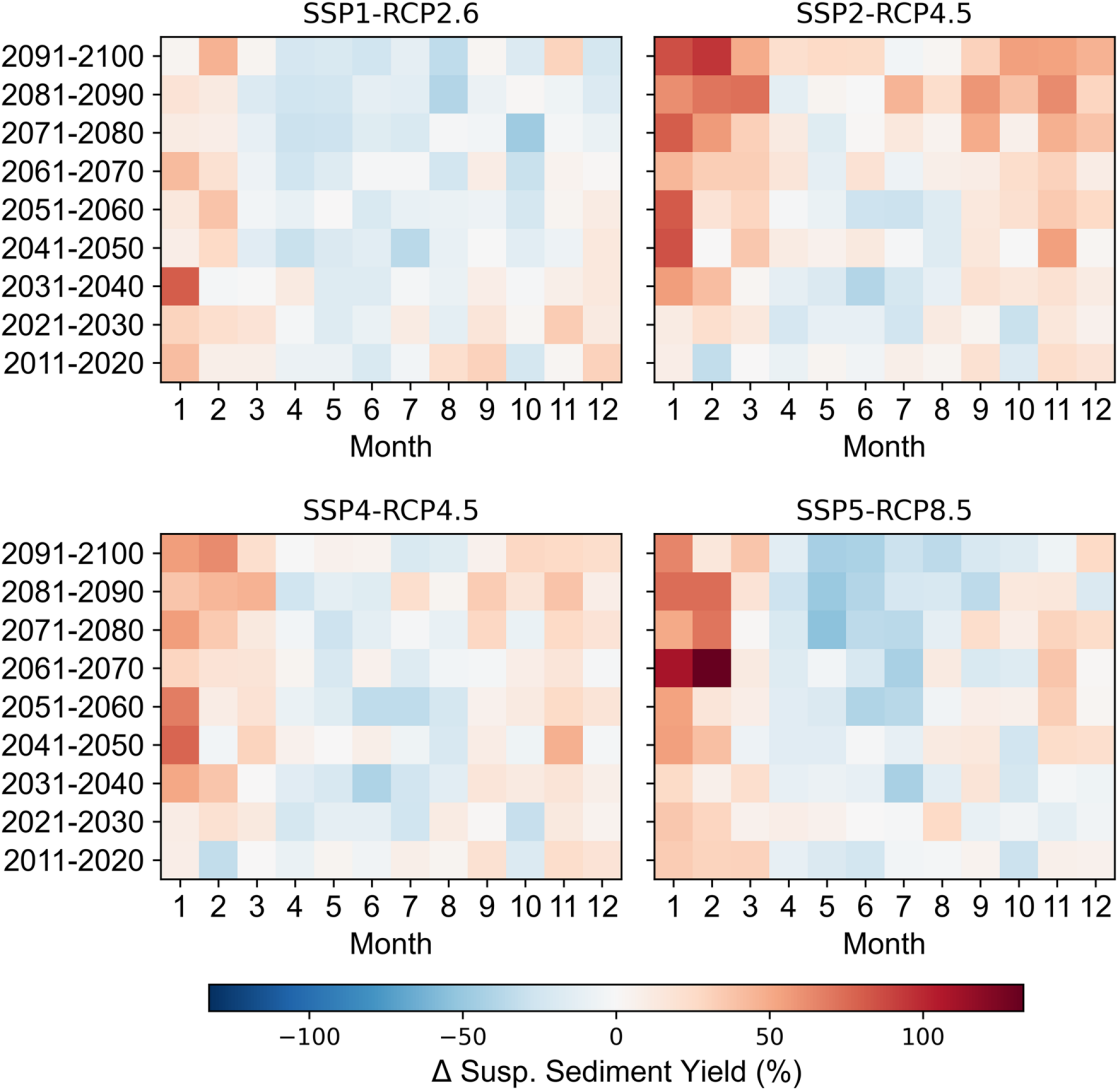
Decadal percent changes relative to the reference period (1981–2010) of the mean monthly change in the suspended sediment yield, SSY (%), of the Mediterranean Devoll catchment for the four global change scenarios investigated. The figure was created by the authors using Matplotlib 3.5.1 (https://matplotlib.org/).

The application of projected land use change in addition to climate change projections amplifies the differences in future predictions for the SSY (Fig. 8). The only global change scenario with a decreasing SSY trend for the Mediterranean Devoll catchment is SSP1-RCP2.6 with − 3% for the mid and − 8% for the far future (Supplementary Information, Table S9). The SSY increases the most for SSP2-RCP4.5 in the mid (21%) and the far (41%) future. SSP4-RCP4.5 leads to a slightly lower increase in SSY in the mid (12%) and far (19%) future. SSP5-RCP8.5 causes a peak increase in SSY of 13% in the mid future, while SSY increases by 8% in the far future. The SSY seasonality is not affected by land use change and is solely driven by hydro-climatic variables, which is why all four scenarios show a decrease in spring and summer and an increase in winter.

**Figure 9:**
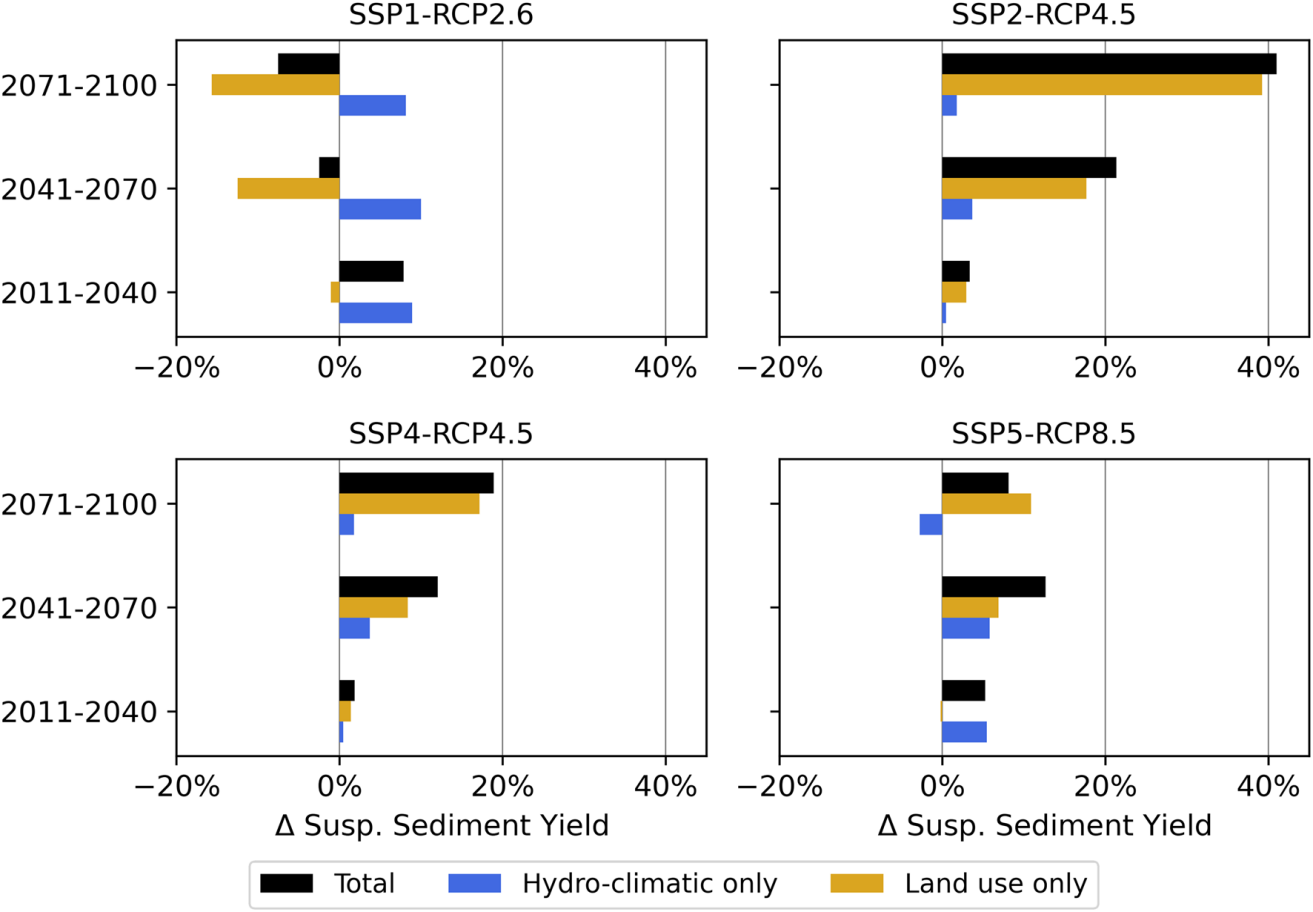
Influence of global (land use and hydro-climatic) change on the SSY (%) of the highly erosive Mediterranean catchment of the Devoll River for future 30-year periods.

The isolated effects of hydro-climatic and land use change on the SSY of the Devoll catchment reveal that land use exerts a continuous influence resulting in either a steady decrease (e.g., SSP1-RCP2.6, see Fig. 9) or increase (e.g., SSP2-RCP4.5, see Fig. 9). Hydro-climatic change does not exhibit such a continuous change pattern. For example, the influence of hydro-climatic change on the SSY for SSP1- RCP2.6, SSP2-RCP4.5, and SSP4-RCP4.5 peaks in the mid future and decreases in the far future. For SSP5- RCP8.5, the trend changes completely in the far future, and hydro-climatic changes only result in a lower annual SSY compared to the reference period due to a decrease in precipitation.

**Figure 10:**
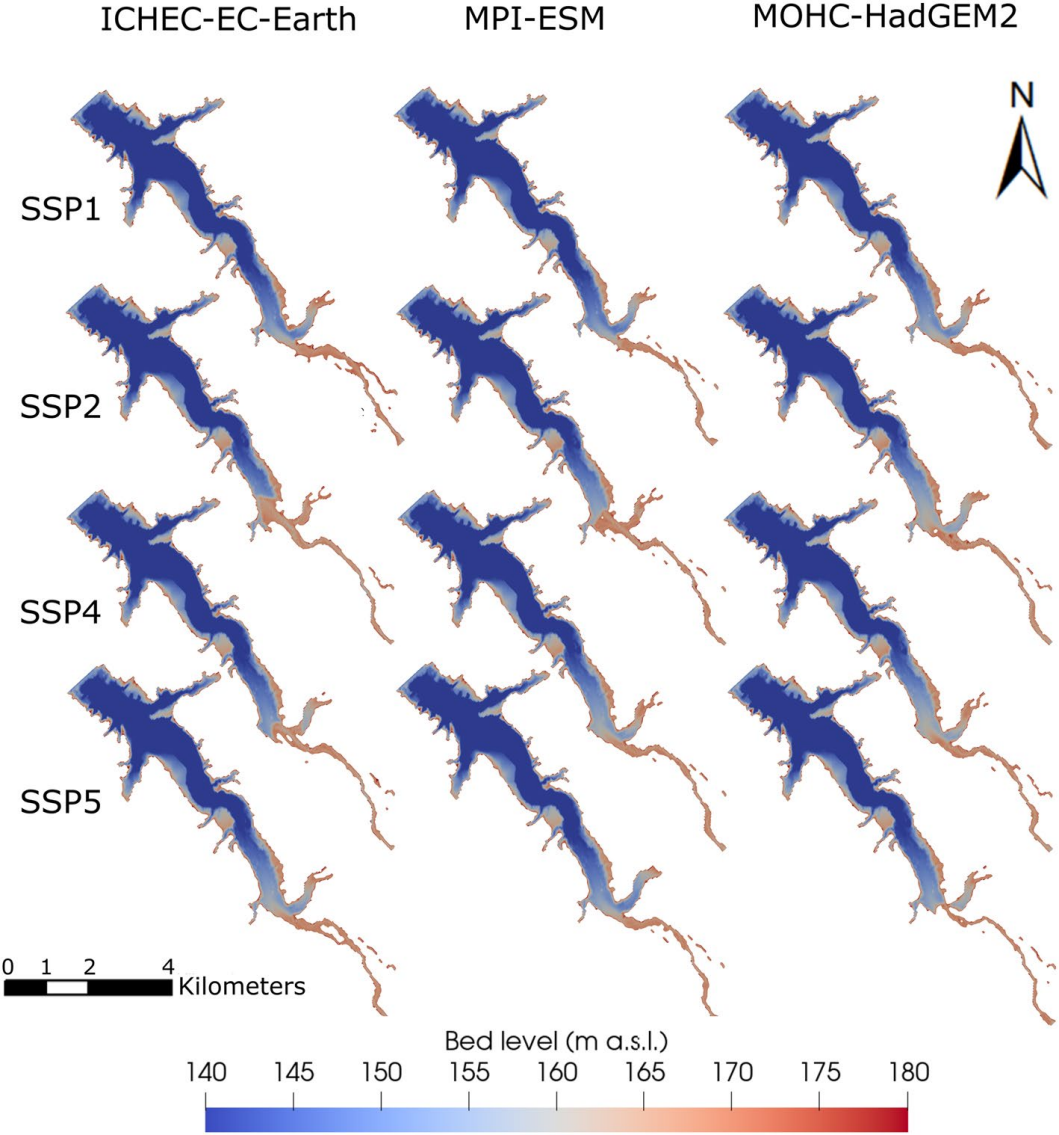
Comparison of reservoir bed levels across four SSPs and three climate models after 84 years of operation (2100).

The considerable changes in the SSY effect on sedimentation processes in the reservoir. Among the global change scenarios, the greatest volume loss is observed for SSP2-RCP4.5, while SSP1-RCP2.6 leads to the lowest loss (Supplementary Information, Fig. S8a). The differences between the global change scenarios are much more significant than those observed among isolated climate change scenarios. Similar to the climate change scenarios, the TE decreases across all SSPs over 84 years of impoundment, declining from initial values exceeding 99% to values ranging between 96.3 and 98.6% (Supplementary Information, Fig. S8b). Notably, no statistically significant differences in TE were identified among the SSPs, while the deposition patterns point to considerable delta formation and progression for SSP2, and less pronounced, SSP4. Specifically, these two scenarios with high SSY cause the delta to advance up to 4.5 km into the reservoir after 84 years of operation (Fig. 10). In contrast, for SSP1, the scenario with the lowest SSY, the delta does not reach the eastern tributary. Furthermore, scenarios with higher storage losses and consequently lower storage volumes tend to exhibit lower TEs.

## Discussion

The model chain was applied to the Mediterranean Devoll catchment, serving as a representative example of regions characterized by high erosion rates and a Mediterranean hydro-climatic pattern. Temperature and precipitation trends aligned with studies conducted in other Mediterranean areas and impact both discharge and sediment production within the catchment^1,28,45,71^. While the average precipitation patterns vary marginally for RCP4.5 and the average decreases for RCP8.5, the seasonal variations for these pathways without emission reduction are projected to intensify with less precipitation and discharge in spring and summer. Projected increases in winter precipitation are unlikely to be stored as snow due to significant temperature rises across all RCPs. As a result, the elevated winter precipitation will be less available during the even drier spring and summer months, which are crucial for plant growth and agriculture^72^. Consequently, Mediterranean regions are expected to require increased artificial water storage, irrespective of the ultimately adopted RCP. Furthermore, more sediment will be mobilized due to less snow and higher peak flows in winter appearing nearly one month earlier in the year^1,73^, which will increase reservoir sedimentation in Mediterranean regions.

Reservoir sedimentation is controlled by the sediment yield and discharge coming from the catchment. Seasonal changes in the sediment yield show an increase in the winter months and a decrease in spring and summer. However, the changes in the sediment yield may not necessarily be proportional to changes in precipitation and discharge^30,74^. Despite considerable reductions in discharge for RCP4.5 and 8.5, the sediment yield either increases or remains within the margin of error of the model chain, providing evidence of rising sediment concentrations, particularly in high emissions scenarios. The higher sediment yields occur despite reduced discharge, which can be attributed to more frequent extreme precipitation events and winter rainfall (instead of snow) on less vegetated soils, which are more susceptible to erosion. Consequently, more erosive rainfall affects erodible soils, especially in the most likely and less sustainable climate change scenarios of RCP4.5 and RCP8.5. The consideration of the decreasing share of grassland and forest in SSP2-RCP4.5 and SSP4-RCP4.5 leads to an even more pronounced increase in soil erosion and sediment yield. Thus, unsustainable development in Mediterranean catchments leads to higher soil erosion and consequently soil loss, which threatens the livelihood of large portions of the population^75^. Particularly for SSP4-RCP4.5, a vicious circle risks opening up, since global inequality might lead to further environmental degradation and thus even more soil loss^76^. Land use change in the SSP1-RCP2.6 sustainability scenario results in a trend reversal and the sediment yield decreases due to the expansion of grasslands and forests despite increasing precipitation.

In the Mediterranean Devoll catchment, the impact of land use change on annual sediment yield outweighs the effect of hydro-climatic changes, particularly in the far future (Fig. 9). The crucial importance of land use on erosion and sediment yield is consistent with previous findings^22,25,33,77^. For example, alterations in land use resulting from climate change were shown to have a more pronounced effect on soil erosion than changes in precipitation or temperature alone^78^. Therefore, effective management of local land use represents an opportunity to alleviate the effects of climate change^79^. Actions such as afforestation^78^, contour farming, or riparian buffers can effectively reduce sediment yields and subsequent reservoir sedimentation. In cases where bioenergy production requires acreage (e.g., SSP2), the considerate selection of bioenergy crops can help regulate sediment production in a catchment^80^. Preferably, the cultivation of perennial grasses with extensive vegetation cover should be favored over grain-based energy crops, such as soybean, corn, and rapeseed^81^. However, not all sediment production processes are directly driven by land use. For instance, fluvial erosion is affected by bank stability and the basal shear stress of a river.

While projections for the Devoll catchment indicate that land use change will dominate over the effects of climate change on soil erosion and suspended sediment yield in Mediterranean areas, contrasting results are expected in regions with substantial increases in rainfall erosivity^47^. For instance, in central Europe or along the North American East coast, hydro-climatic change is likely to exert a greater influence on soil erosion than changes in land use.

Reservoirs can mitigate seasonal hydrological fluctuations caused by global change, provided that sediment inflow does not substantially diminish their storage capacity. However, storage capacity is expected to decrease as sedimentation rates increase, which in turn will affect, for example, the availability of water for irrigation. To this end, sustainable reservoir operation should aim for a small trapping efficiency, which naturally declines over time due to narrower cross-sections with higher sediment conveyance capacity through increased mean flow velocities^42^. In the Banja reservoir, a typical decrease in TE is observed (Fig. 6), especially in scenarios characterized by high sediment yield. However, the high uncertainty in the climate projections hampers a clear differentiation between TE trends among RCPs and SSPs. Due to higher flow velocities and transport capacities in the reservoir, TE decreases primarily when high discharges occur over a period of several weeks, while sediment originating from stochastically occurring singular extreme events tends to be largely trapped. Beyond TE, the predicted reservoir bed levels (Fig. 10) indicate considerable sedimentation at the reservoir head (delta deposition), leading also to the formation of a channel. This process of channel formation is a common characteristic observed in large reservoirs^42,82^. The most significant difference among the global and climate change scenarios is that the channel and the delta progression extend further downstream in scenarios with high sediment yield (e.g., RCP2.6, SSP2-RCP4-5, and SSP4-RCP4.5).

These findings provide valuable insights for implementing targeted reservoir management strategies. For example, one option to reduce significant upstream sediment deposition is to lower the water level before the anticipated high sediment inflow during the wet season. This approach allows sediments to be re-suspended and transported closer to the dam, with the option of routing them through the reservoir or storing them in the dead storage. However, these sediment deposits near the dam can pose safety concerns, such as blockage of bottom outlets, while offering opportunities for easier flushing that facilitates reservoir management^7^. Thus, the precise 3d modeling also aids in delineating dam safety concerns, but the model chain cannot yet process feedback of decreasing reservoir storage on water availability and thus on land use.

Although this study did not consider specific management strategies and the monitoring period was limited, it emphasizes the capacities of a novel interdisciplinary model chain to predict long-term reservoir sedimentation in Mediterranean areas. Still, absolute sediment-related quantities are subject to considerable uncertainty, primarily stemming from variations in climate projections and their propagation through the model chain. On the contrary, the lower uncertainty because of the physically greater precision of the 3d numerical model played a subordinate role. Thus, to address the uncertainty in long-term predictions of reservoir sedimentation, a primary concern will be to improve the accuracy of climate predictions. Ultimately, the climate projections only determine the framework conditions of this inherently precise and efficient model chain, which produces predictions of reservoir sedimentation with unprecedented precision and time horizons to test and implement effective land-use management actions, even in remote regions.

## Conclusions

A complex interdisciplinary and physics-informed model chain demonstrates considerable impacts of hydro-climatic and land use changes on water availability, sediment production, and reservoir sedimentation in a Mediterranean region Applied to the Devoll catchment, a typical Mediterranean mountainous region with high sediment production, the model chain shows that global change leads to increased sediment yields and decreased river discharge, with seasonal shifts for most of the climate and land use change projections considered. A low-emission scenario (SSP1-RCP2.6) sustains higher discharges by 2100, while mid to high greenhouse gas emission and unequal development (SSP4-RCP4.5) or fossil-fueled development (SSP5-RCP8.5) scenarios amplify water scarcity. Specifically, increased winter rainfall, reduced snowfall, and decreased summer precipitation contribute to limited water availability during hot and dry Mediterranean summers, emphasizing the need for artificial water storage in reservoirs.

In the low-emissions scenarios, higher discharges lead to elevated sediment yields but lower sediment concentrations compared to less sustainable emissions scenarios. In particular, the sediment concentration decreases with the implementation of sustainable land use (SSP1-RCP2.6). In contrast, less sustainable land use leads to higher sediment concentrations and sediment yields (SSP2-RCP4.5 and SSP4-RCP4.5) due to decreased forest and grassland areas. The scenarios with higher sediment yields experience the most substantial loss of storage volume and the delta moves further downstream, resulting also in a decrease in trapping efficiency (TE).

The three-dimensional (3d) hydro-morphodynamic model at the end of the model chain goes beyond simplistic parameters, such as TE, and provides valuable insights into sedimentation patterns and processes controlled by global change. Although simplistic models may yield similar TE results as multidimensional numerical models, a spatially explicit 3d model pinpoints hotspots of sedimentation, providing crucial information for sustainable reservoir management practices like dredging or reservoir flushing.

Ultimately, the interdisciplinary model chain highlights that land use change outweighs climate change effects in Mediterranean regions. Therefore, localized management actions for land use change in the catchment, such as policy-enforced crop adaptations and afforestation, can reduce soil loss and sediment production. In addition, the long-term prediction strength of the model and the spatially explicit deposition patterns enable the implementation of targeted reservoir management strategies.

### Supplementary Information

Supplementary Information.

## Data Availability

The complete datasets generated during and/or analyzed during the current study are available from the corresponding author on reasonable request.
