# Supplementary material for: An interdisciplinary model chain quantifies the footprint of global change on reservoir sedimentation
Source: Sci Rep. 2023 Nov 17;13:20160. doi: 10.1038/s41598-023-47501-1 (PMC10656515; doi:10.1038/s41598-023-47501-1)
Supplement: Supplementary file 1 — Supplementary Information. [file 41598_2023_47501_MOESM1_ESM.docx]

Supplementary Information for

**An interdisciplinary model chain quantifies the footprint of global change on reservoir sedimentation**

(Scientific Reports)

**Corresponding author:** Kilian Mouris

# SI 1 Climate projections

Three Global Climate Models (GCMs), which were dynamically downscaled by two different Regional Climate Models (RCMs) were used to estimate the impact of climate change on reservoir sedimentation (Table S1). For more comprehensive information regarding the Global Climate Models (GCMs) utilized in this study, readers are directed to the following references: Popke et al.^1^ for MPI-M-MPI-ESM-LR, Hazeleger et al.^2^ for ICHEC-EC-EARTH, and Collins et al.^3^ for MOHC-HadGEM2-ES. The regional climate models employed in this research are described in Kjellström et al.^4^ for SMHI-RCA4 and van Meijgaard et al.^5^ for KNMI-RACMO22E_v2.

**Table S1.** GCM-RCM combinations used to analyze climate change impacts.

| GCM | RCM |
| --- | --- |
| ICHEC-EC-EARTH | SMHI-RCA4_v1a |
| MPI-M-MPI-ESM-LR | SMHI-RCA4_v1 |
| MOHC-HadGEM2-ES | KNMI-RACMO22E_v2 |

A more detailed overview of the emissions scenarios studied, including their development, purpose, and interpretation, can be found in van Vuuren et al.^6^ for the Representative Concentration Pathways (RCPs) and in Riahi et al.^7^ for the Shared Socioeconomic Pathways (SSPs).

# SI 2 Hydrological modeling

Table S2 summarizes the main sub-processes involved in WaSiM and the selected methods for obtaining the values for each of them.

**Table S2.** Main sub-processes and approaches selected for the WaSiM simulations.

| Sub-processes | Selected method(s) | Comments |
| --- | --- | --- |
| Temperature, precipitation and relative humidity interpolation | IDW + EDRINT(a) | Linear combination of IDW and EDRINT |
| Wind speed and global radiation interpolation | IDW | - |
| Evapotranspiration | Penman-Monteith approach for ETP(b) | ETR(c) estimated from actual soil water content and actual capillary pressure |
| Snow accumulation and snow melt | Energy balance approach | The fraction of snow on the total precipitation is given by a threshold temperature |
| Interception | Includes a bucket canopy model | Snow accumulation and melt is computed for both canopy and the surface below |
| Soil model | Richards approach | Model of fluxes within the unsaturated soil zone |
| Groundwater model | Integrated conceptual approach | Baseflow is generated as exfiltration from the groundwater system into the river surface system |
| Routing model | Kinematic wave approach + single linear storage | Translation is determined from Manning’s formula; single reservoir to account for retention |

^(a)^ IDW = Inverse distance weight method, EDRINT = Elevation dependent regression with internal pre-processing

^(b)^ ETP = Potential evapotranspiration

^(c)^ ETR = Real evapotranspiration

The calibration of the model, carried out for the period between May 2016 and April 2018, was based on measured discharge data at the Kokel gauging station. In total, 11 parameters were adjusted during the calibration, whereby 5 proved to be less clearly identifiable than the others. To evaluate the performance of the calibration, different criteria were used. The model calibration resulted in a Root-mean-square error (RMSE) of 0.09 m 3h^-1^, an R-squared (R²) of 0.78, a Nash-Sutcliffe efficiency (NSE) of 0.72, a Kling-Gupta efficiency (KGE) of 0.82 and a Percent Bias (PBIAS) of 8.86. An additional validation was carried out using monthly discharges from the Global Runoff Data Centre for the period 01/1980 to 12/1984 leading to a RMSE of 0.59 mm d^-1^, an R² of 0.65, a NSE of 0.58, a KGE of 0.71 and a PBIAS of 18.98. Ideally, PBIAS and RMSE would be equal to 0, and NSE, R², and KGE would be equal to 1. Better performance for the validation was not to be expected as the Devoll River's discharge was subject to strong anthropogenic influences and, until 2000, the river was frequently diverted into Lake Prespa during the rainy season.

# SI 3 Soil erosion and sediment transport model

The soil loss A (t ha^− 1^ yr^− 1^) was calculated as the product of six erosion risk factors (Eq. (1)). Table S3 describes briefly the individual factors and their calculation.

|  | $A = R\cdot K\cdot C\cdot L\cdot S\cdot P$ | (1) |
| --- | --- | --- |

**Table S3.** Erosion risk factors and their calculation.

| Factor | (Units) | Description |
| --- | --- | --- |
| R | (MJ mm (ha h yr)^-1^) | Rainfall-runoff erosivity factor calculated from projected precipitation data according to Diodato and Bellocchi^8^ and Mouris et al.^9^ |
| K | (t ha MJ^-1^ mm^-1^) | Soil erodibility factor according to Wischmeier and Smith^10^ |
| C | - | Cover and management factor based on land cover^11^ and projected land use changes^12^ |
| L, S | - | Slope length and slope steepness factors represent the effect of topography on soil erosion according to Zhang et al.^13^ |
| P | - | Support practice factor expresses the influence of contouring on soil erosion (only applies to agricultural land) |

K, LS, and P factors are constant and do not change over time within the scenarios (i.e., not influenced by global change). The K factor is calculated as a function of soil structure, organic matter content, soil texture, and soil permeability according to Wischmeier and Smith^10^. A correction factor was implemented to account for the reduced soil erodibility in the presence of cobbles^14^, and all required soil data were obtained from the European Soil Database^15^ and the Harmonized World Soil Database^16^. This study calculates the LS Factor by employing a multi-flow direction algorithm, which incorporates slope, aspect, and downhill flow direction^13^. This approach accounts for flow convergence based on the contributing surface and slope cutoff conditions^17^. The effectiveness of contouring on soil erosion depends on the slope of the agricultural land. Tilling and planting along contours that are perpendicular to the overland flow decreases the runoff velocity and leaves more time for infiltration. Contouring proves highly efficient on slopes ranging from 3% to 8% where it can achieve erosion reduction rates of up to 50%. However, in flat terrain or regions with slopes exceeding 25%, the effectiveness of terracing is significantly diminished^18,19^.

Only the R and C factors were considered to be affected by global change. We calculated the R factor at a monthly resolution by taking into account precipitation, temperature, and snow cover derived from the hydrological model. The equations used to determine the R factor remained unchanged for predicting global change scenarios because there were no discernible trends in the erosivity^20^ and intensity^21^ (e.g., annual maximum five-day precipitation or extreme precipitation totals) of precipitation events for the study area. Beyond the seasonal variability of the C factor, we also considered projected land use changes under different global change scenarios. Thus, the C factor was calculated for each pixel based on the pixel-specific land use percentages. Figure S1 shows the evolution of the catchment’s mean C factor for the investigated global change scenarios. A higher proportion of natural grassland and forest resulted in smaller C factor values, while an increase in cropland led to higher C factors. The seasonal C factors per land cover class are shown in SI Table S4. Two distinct model setups were utilized, with slightly differing characteristics. The projected land cover changes^12^ (used for the SSP-RCP scenarios) were represented at a coarser spatial resolution, comprising 8 classes, whereas the Corine land cover^11^, used for the RCP scenarios, encompassed 21 classes for the study area. Hence, the two model setups were calibrated separately to represent the observed suspended sediment concentrations at the monitoring station.


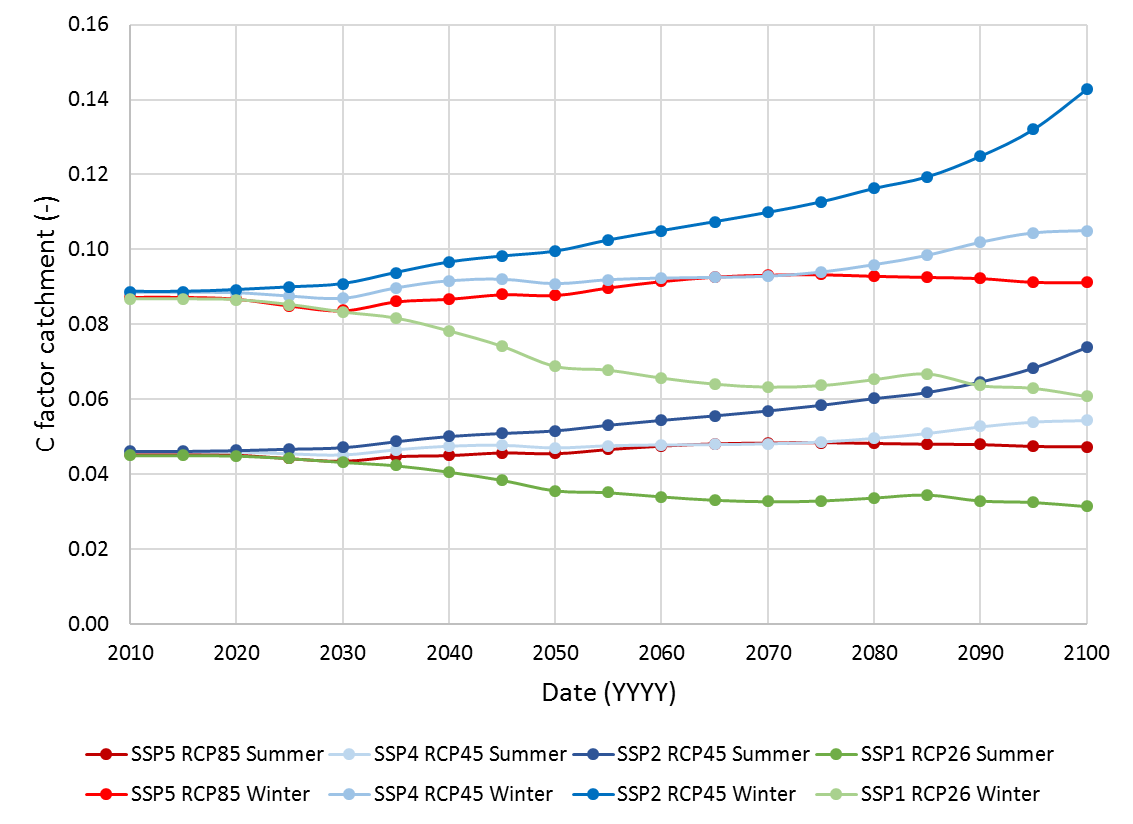


**Figure S1.** Evolution of the mean C factor of the Devoll catchment for the investigated global change scenarios.

**Table S4.** Seasonal C factor values used for the observed and the projected land cover classes.

| Land cover class | | C factor value (-) | |
| --- | --- | --- | --- |
| Observed | **Projected** | Summer | Winter |
| Discontinuous urban fabric | Urban | 0.000 | 0.000 |
| Industrial or commercial units |  | 0.000 | 0.000 |
| Road and rail networks and associated land |  | 0.000 | 0.000 |
| Mineral extraction sites | - | 0.750 | 0.750 |
| Green urban areas | - | 0.100 | 0.100 |
| Vineyards | - | 0.237 | 0.363 |
| Fruit trees and berry plantations | Agriculture | 0.165 | 0.235 |
| Pastures | Grass (C4) | 0.087 | 0.113 |
| Broad-leaved forest | Needleleaf and Broadleaf forest | 0.0008 | 0.0023 |
| Coniferous forest |  | 0.0008 | 0.0023 |
| Mixed forest |  | 0.0008 | 0.0023 |
| Natural grasslands | Grass (C3) | 0.032 | 0.058 |
| Moors and heathland | - | 0.037 | 0.073 |
| Sclerophyllous vegetation | - | 0.042 | 0.068 |
| Transitional woodland-shrub | Broadleaf shrub | 0.017 | 0.036 |
| Beaches, dunes, sands | - | 0.750 | 0.750 |
| Bare rocks | - | 0.000 | 0.000 |
| Sparsely vegetated areas | - | 0.248 | 0.302 |
| Inland marshes | - | 0.006 | 0.006 |
| Water bodies | - | 0.000 | 0.000 |
| Arable land | Agriculture | 0.116 | 0.225 |
|  | Bioenergy | 0.116 | 0.225 |

The RUSLE assesses the spatial distribution of gross soil loss, and the SEDD model^22^ is necessary as an additional sediment routing method to estimate sediment yield on a catchment scale. The sediment yield represents the sediment mass passing a defined boundary, such as the outlet of a catchment. The ratio between sediment yield and catchment soil erosion is known as the sediment delivery ratio (SDR). We calculated the pixel-specific SDR_i_ as a function of the travel time along the flow path to the closest river channel. The pixel-specific overland flow velocity depends on the slope and surface roughness, which is implemented according to the land use^9,23^. In addition to calibration, the model was validated using two separate 5-month calibration and validation periods and a complementary leave-one-out cross-validation^9^.

# SI 4 3d hydro-morphodynamic model

SSIIM 2 solves the three-dimensional Reynolds-averaged Navier-Stokes equations (RANS) using a finite volume method for discretization. The model utilizes an unstructured and adaptive grid consisting of cells with a spatial resolution of approximately 50 m x 50 m in the horizontal direction and up to 10 vertical cells in the deepest areas of the reservoir. For sediment transport, SSIIM 2 solves the transient convection-diffusion equations for suspended sediment transport and employs an empirical formula by van Rijn for bedload transport^24,25^. Relevant grain sizes range from 5 µm to 1 mm for the here-featured case of the Banja reservoir. However, the relevant grain sizes are reservoir-specific and should be confirmed by a field survey. To enable efficient simulations of long-term global change scenarios until the year 2100 within a reasonable computing time (3.5 weeks per run using 8 cores, 3.7 - 4.8 GHz), SSIIM 2 utilizes an implicit solver for the Navier-Stokes equations, allowing for the use of large time steps (5400 s for this case).

The transient boundary conditions of the SSIIM2 numerical software are defined in the so-called timei file. Since manual creation of these files is not feasible for long-term simulations such as various climate scenarios, we developed a Python code^26^ that automates the creation of the timei file. The inflow discharges were directly derived from the hydrological model and the sediment concentrations were derived from the soil erosion and sediment transport model. The calculation of the outflow was based on multiple factors, notably the reservoir water level, inflow, reservoir storage curve, and site-specific operation guidelines that account for a desired seasonal water level. To this end, we implemented a monthly target water level that should be met by the operation of the reservoir. Based on the past operating strategy, water levels were highest in May/June and lowest before the wet season in November. The following Table S5 describes the input argument required to run the code and create the timei files.

**Table S5.** Input arguments and their description required to create the timei boundary condition file using the developed python code.

| Input argument | Description |
| --- | --- |
| q_path | File path (PATH/name.b16) where the results from the WaSim results are stored |
| q_storage | File path (PATH/name.txt) where the reservoir’s storage curve is stored |
| sy_folder | Folder path (PATH/folder name) containing the total sediment yield data for each sub-catchment |
| catchment_order | Names of the sub-catchments to be considered, plus the .txt files for each sub-catchment must include the name of the sub-catchment in the file name |
| sediment_density | Sediment density (kg m^-3^) to calculate the volume concentration |
| turbine_capacity | Maximum discharge (m³ s^-1^) that can pass through the turbines |
| time_interval | Integer that indicates the time frequency to use: 0 to keep the input data frequency, 1 for a daily frequency, 2 for a monthly frequency |
| wl_threshold | Target water level for each month. The array must contain 12 water levels (m) |
| target_wl_upper_boundary | The upper boundary relative to the target water level (m) |
| target_wl_lower_boundary | The lower boundary relative to the target water level as a negative integer (m) |
| target_wl_maximum | The maximum water level of the reservoir. An exceedance leads to discharge via the spillway. |
| restrict_timei_date | Restricts the timei output file to the time frame of timei_date_start and timei_date_end if set to true. |
| timei_date_start | The start date of the timei file |
| timei_date_end | The end date of the timei file |
| results_folder | Path of the main result folder |

The operational strategy outlined in Table S6 is implemented to preserve the current water level close to the seasonal target water level. Figure S3 illustrates the resulting water level for a specific timeframe spanning from January 2059 to December 2064 under the climate change scenario MPI RCP 8.5. The diagram at the bottom displays the inflow into the reservoir as well as the outflow from the reservoir through both the turbines and the spillway. Notably, a flood event occurs in January 2060, characterized by an inflow of approximately 325 m³ s^-1^. Given that this inflow surpasses the capacity of the turbines (approximately 108 m³ s^-1^), the water level rises to the maximum level, and the excess water is discharged via the spillway. Conversely, during a flood event in January 2063, the water level remains below the maximum level, enabling control through turbine operation. In the initial months of 2061, the wet season experiences low inflows, resulting in a turbinated discharge that falls short of the inflow necessary to maintain the target water level. It is important to note that the operational strategy employed is fictitious and does not consider factors such as energy prices or the current operating rules of the hydropower operator. Nonetheless, it emulates realistic behavior and facilitates the study of long-term sedimentation processes within the reservoir.

**Table S6**. Reservoir operation strategy used to create the boundary conditions file to simulate long-term reservoir sedimentation.

| Water level | Outflow | Description |
| --- | --- | --- |
| current_wl < target_wl + target_wl_lower_boundary | Turbine = 0, Spillway = 0 | All inflowing water is stored in the reservoir |
| target_wl + target_wl_lower_boundary < current_wl < target_wl | Turbine = Inflow * 0.5 (≤ turbine_capacity), Spillway = 0 | The turbine runs at half the inflowing discharge, but never more than the maximum turbine capacity |
| target_wl < current_wl < target_wl + target_wl_upper_boundary | Turbine = Inflow (≤ turbine_capacity), Spillway = 0 | The turbine runs at the inflowing discharge, but never more than the maximum turbine capacity |
| target_wl + target_wl_upper_boundary < current_wl < target_wl_maximum | Turbine = turbine_capacity, Spillway = 0 | The turbine runs at full capacity |
| target_wl_maximum ≤current_wl | Turbine = turbine_capacity, Spillway = Inflow - Turbine (≥ 0) | The turbine runs at full capacity (turbine_capacity) and all excess inflow is released through the spillway |


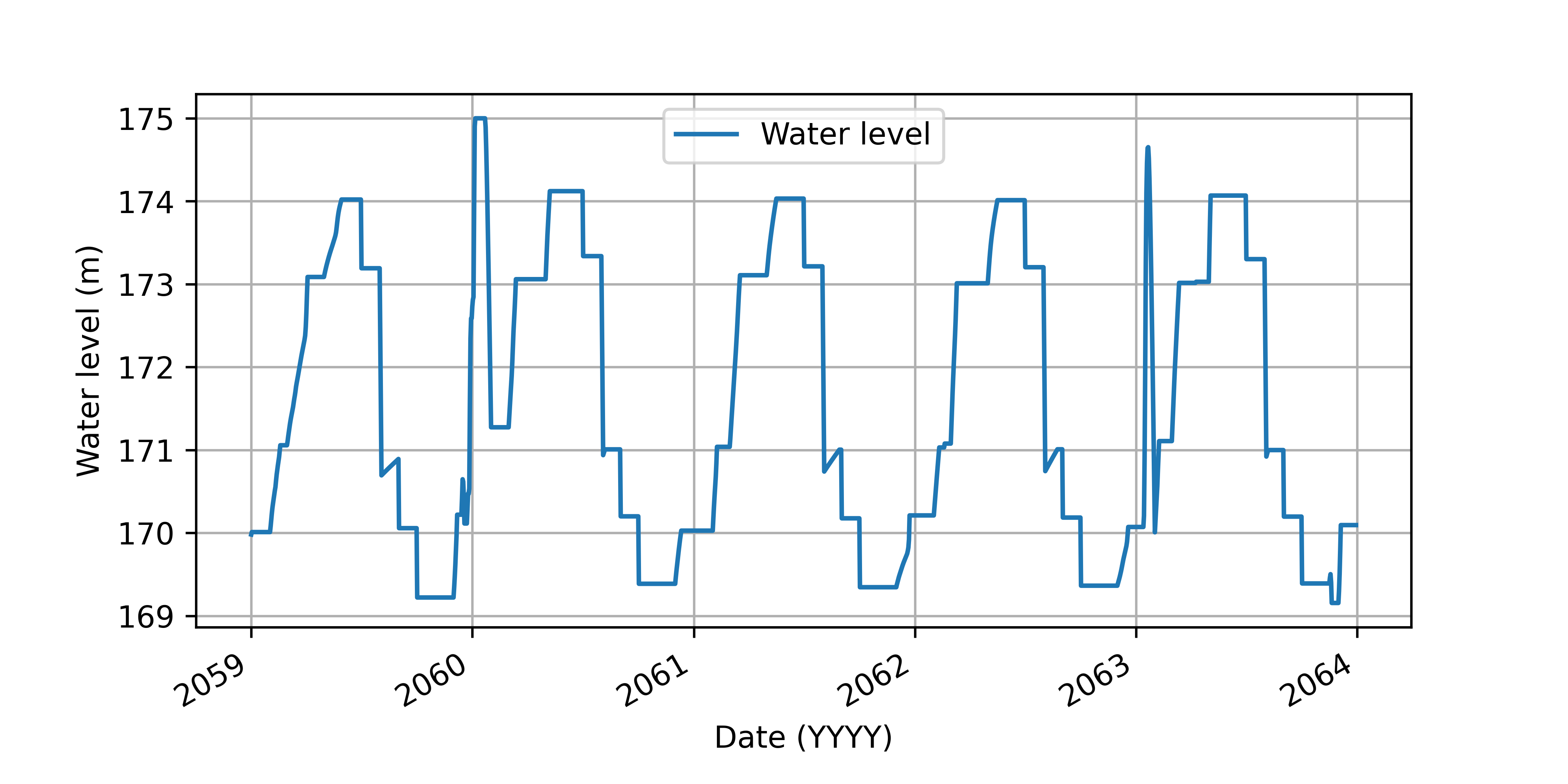


**Figure S2.** Water level of the reservoir for an exemplary period from January 2059 to December 2064 for the climate change scenario MPI RCP 8.5.


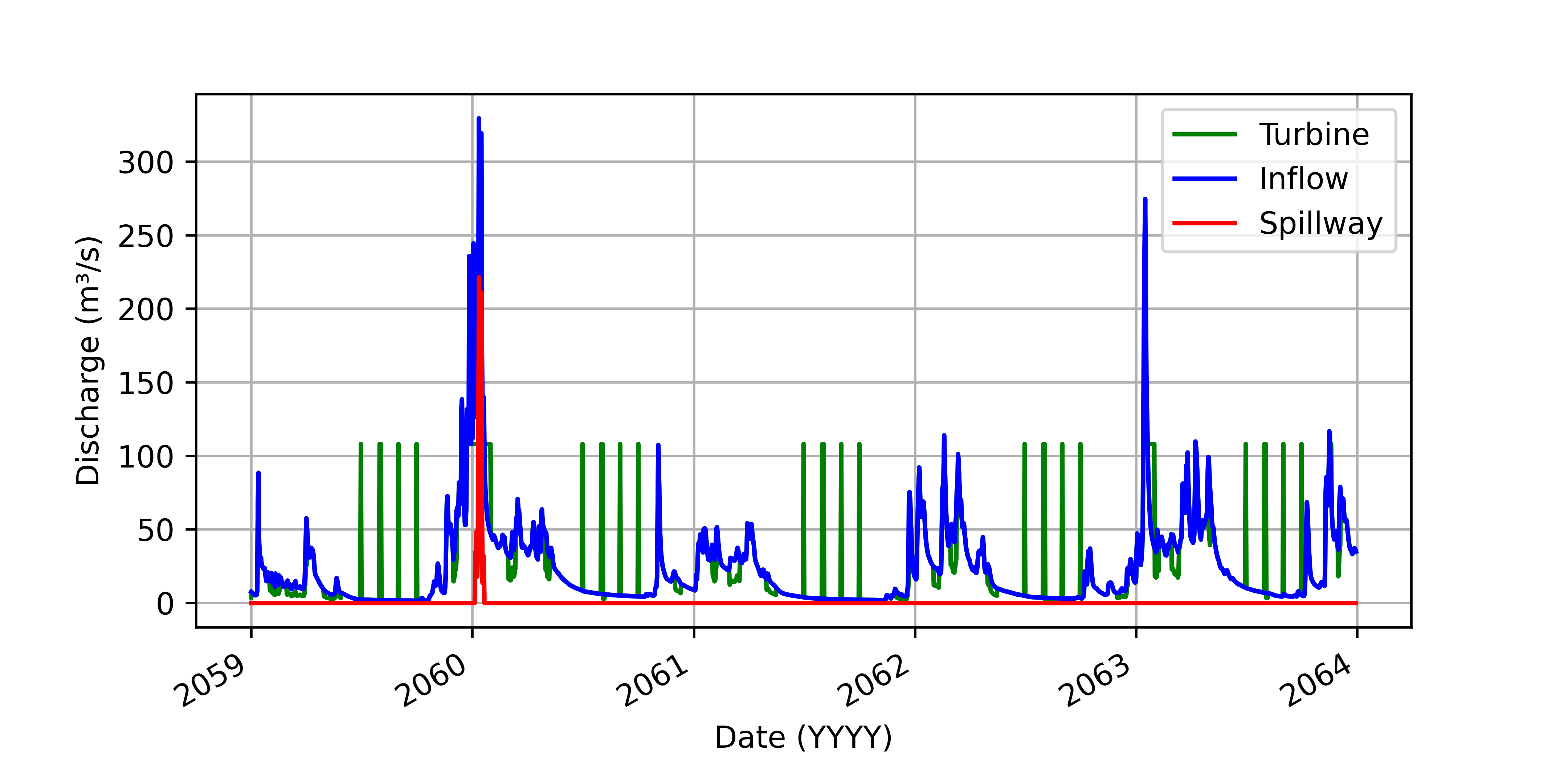


**Figure S3.** The inflow into the reservoir in blue, the outflow through the turbines in green, and the spillway outflow in red.

To facilitate the use of larger time steps in the 3d simulation, we increased the thickness of the upper active sediment layer to 0.30 m. This value significantly exceeds the default thickness associated with the maximum sediment grain size. The purpose of this adjustment was to allow for erosion when the water level decreased. Without this modification, the water level would decrease at a faster rate than the maximum erosion rate, resulting in a grid split when implementing the wetting and drying algorithm. The settling velocity for particles larger than 0.1 mm was determined using the Ahrens method^27^, while Stokes law was used for smaller particles. Nevertheless, the settling velocity of cohesive sediment particles is uncertain due to the influence of flocculation processes. Consequently, the settling velocities of cohesive particles were modified during the calibration.

# SI 5 Additional results – Figures


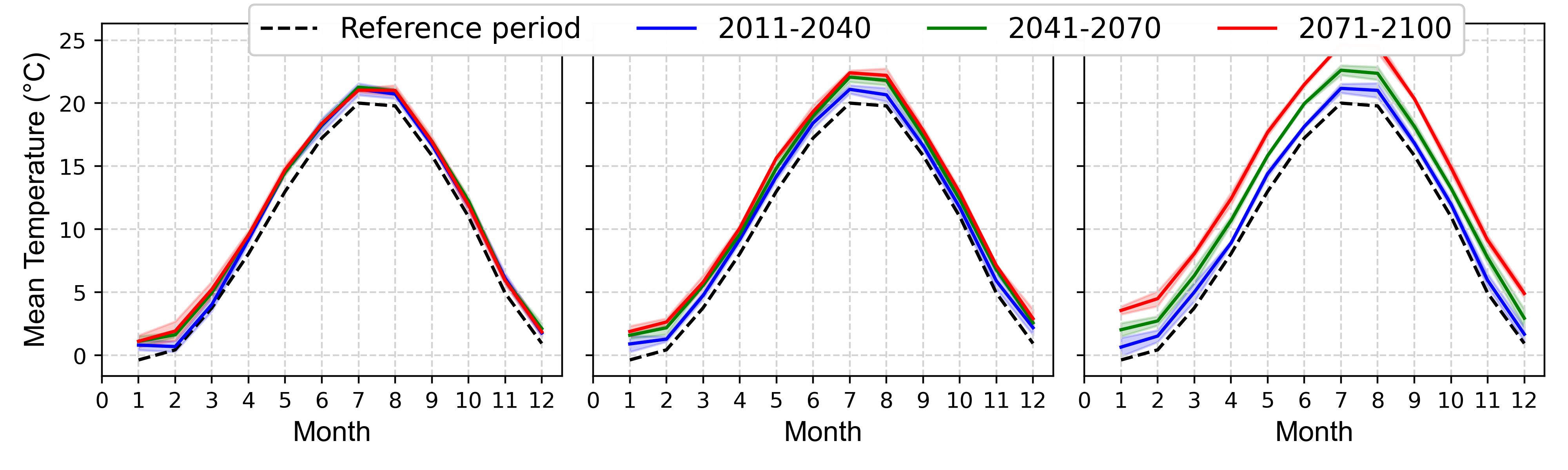


**Figure S4**. Changes in the mean monthly temperature of the Devoll catchment. The shadowed areas represent the spread of ensemble climate projections, calculated as the mean value ± standard deviation.


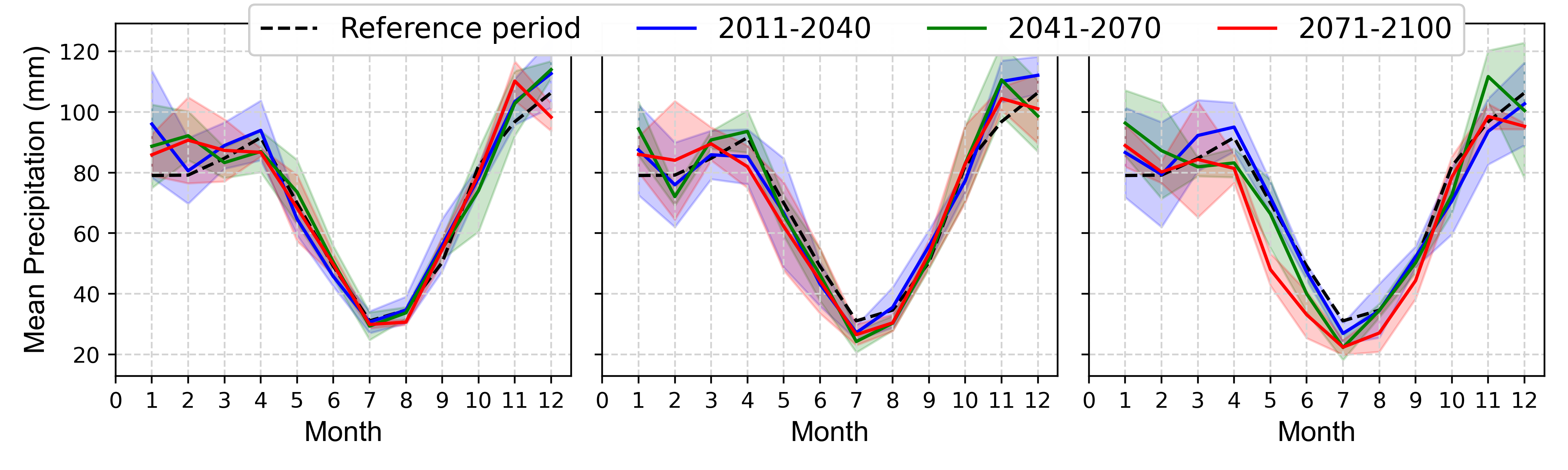


**Figure S5.** Changes in mean monthly precipitation of the Devoll catchment. The shadowed areas represent the spread of ensemble climate projections, calculated as the mean value ± standard deviation.


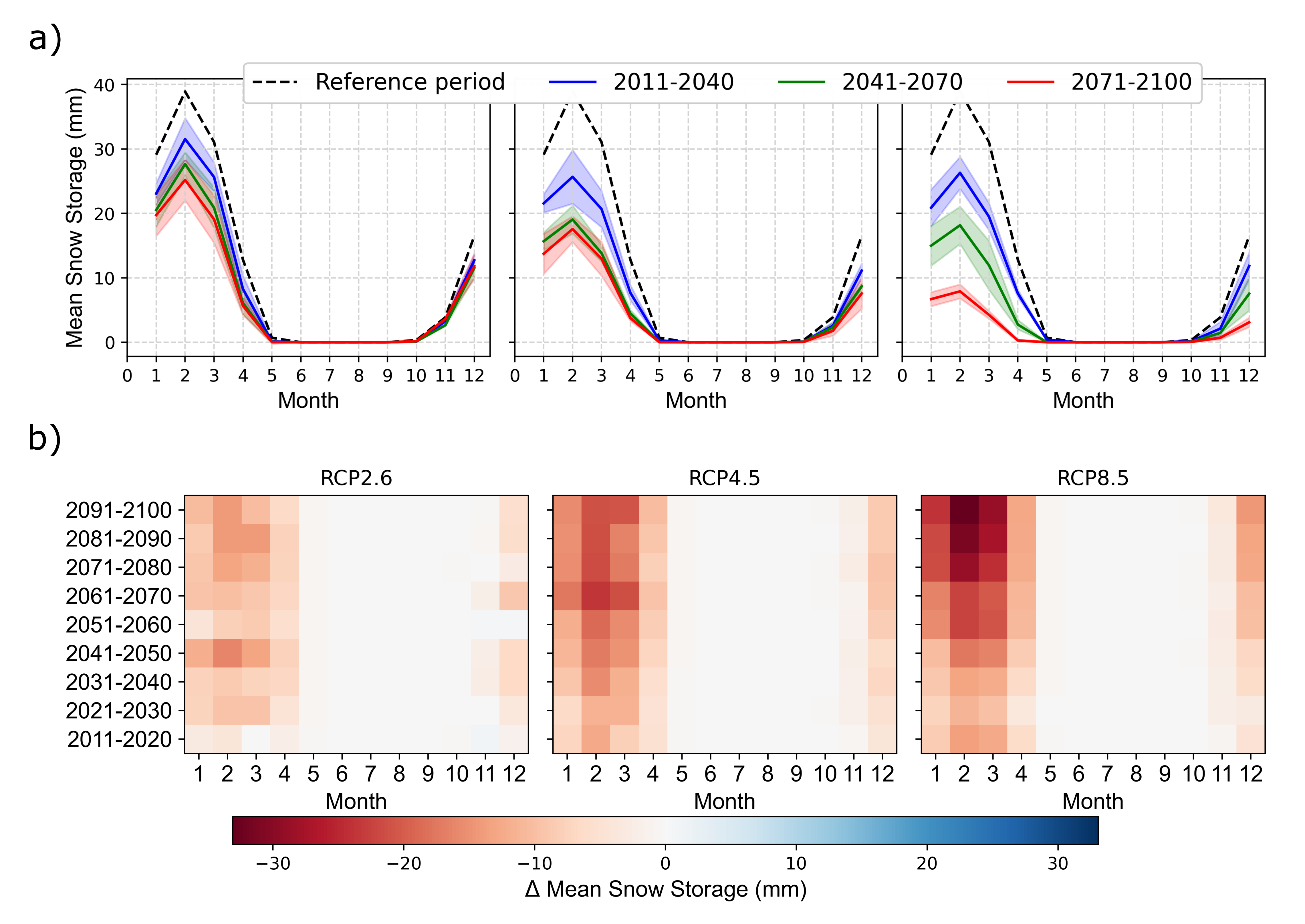


**Figure S6.** Additional results for the mean monthly snow storage in the Devoll catchment. (a) Changes in mean monthly snow storage for three future time periods and (b) decadal changes relative to the reference period in mean monthly and spatial snow storage (mm).


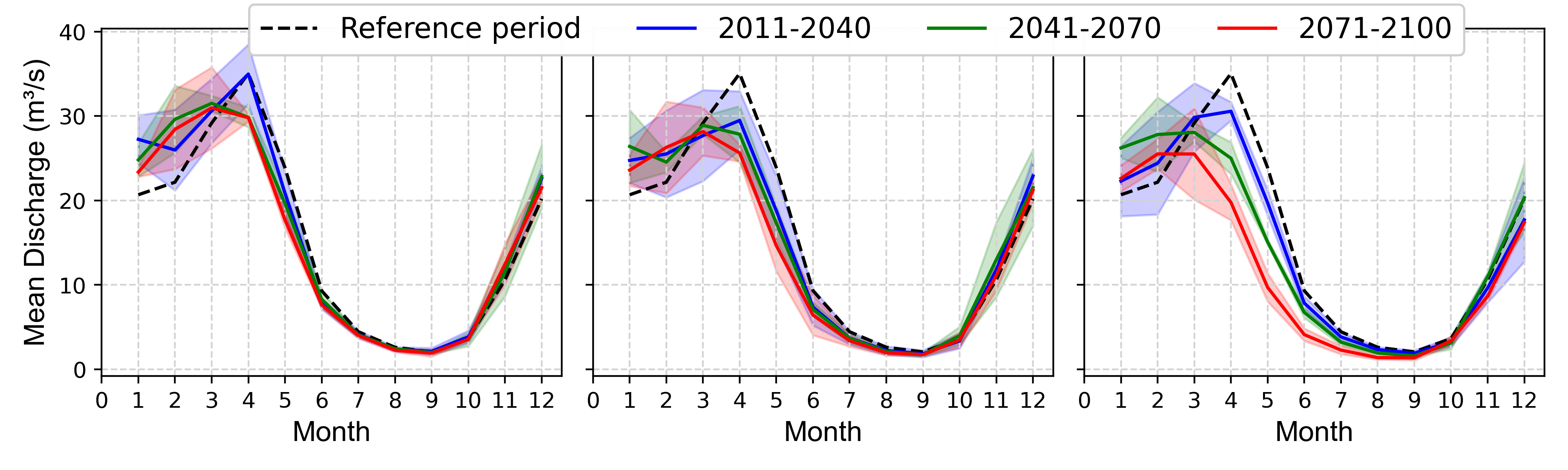


**Figure S7**: Changes in mean monthly discharge of the Devoll River at the Kokel monitoring station. The shadowed areas represent the spread of ensemble climate projections, calculated as the mean value ± standard deviation


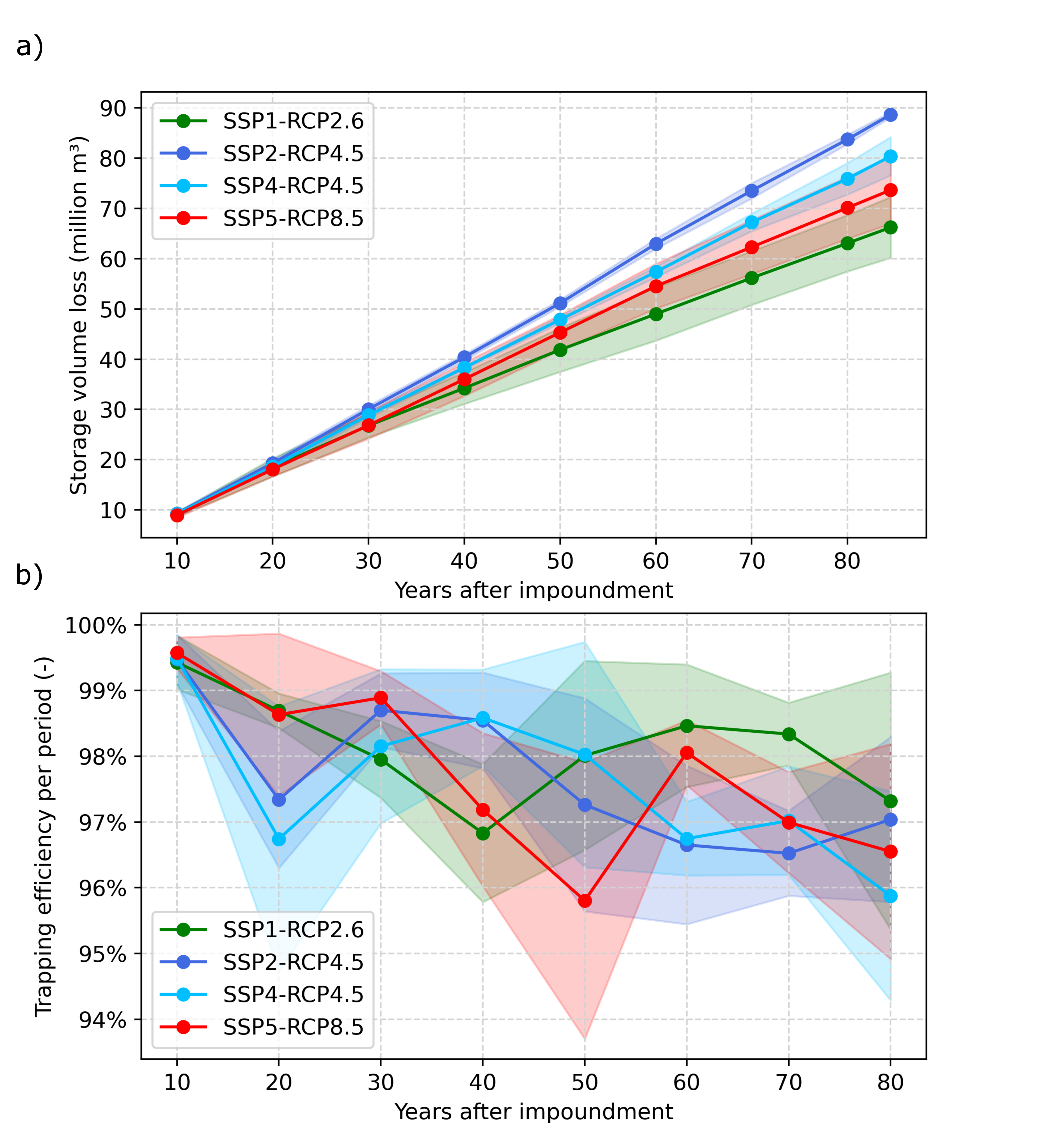


**Figure S8.** Evolution of the loss in storage volume after impoundment (a) and the trapping efficiency at 10-year periods (b) for the Banja reservoir and the four investigated SSPs. The shadowed areas represent the spread of ensemble climate projections, calculated as the mean value ± standard deviation.


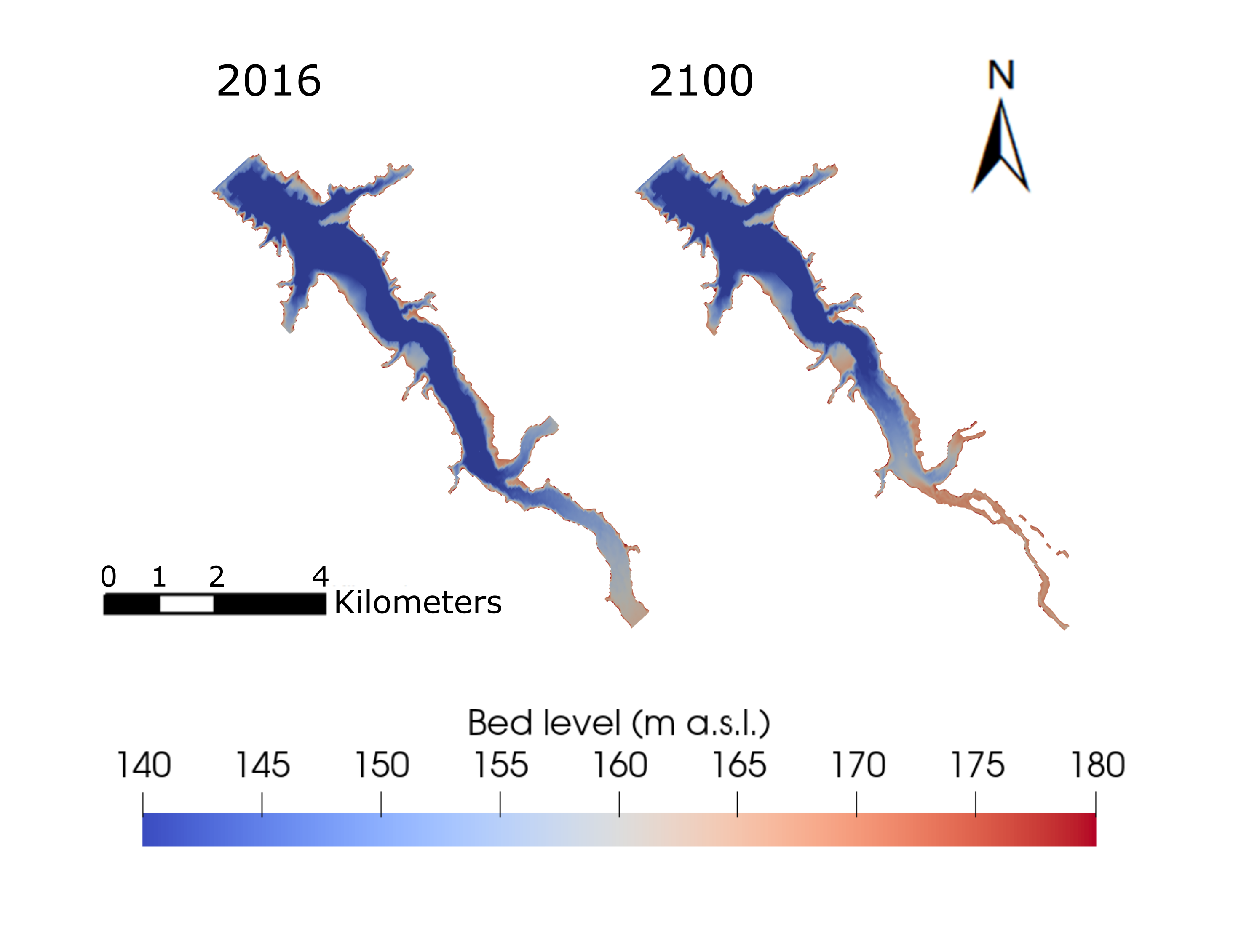


**Figure S9.** Bed levels before impoundment of the reservoir (2016) on the left and after 84 years of operation (2100) on the right. Results shown for SSP5 and the ICHEC-EC-Earth climate model.

# SI 6 Additional results - Tables

**Table S7.** Temperature, precipitation, and snow storage for RCPs 2.6 (green), 4.5 (blue), and 8.5 (red).

|  | Mean value over 30 years until | | | | Changes compared to reference period | | |
| --- | --- | --- | --- | --- | --- | --- | --- |
| Banja catchment | 2010 | 2040 | 2070 | 2100 | 2011-2040 | 2041-2070 | 2071-2100 |
| Temperature | 9.60 °C | 10.54 °C | 10.86 °C | 10.85 °C | 9.8% | 13.0% | 13.0% |
|  | 9.60 °C | 10.63 °C | 11.36 °C | 11.78 °C | 10.7% | 18.4% | 22.8% |
|  | 9.61 °C | 10.66 °C | 12.11 °C | 13.90 °C | 10.9% | 26.0% | 44.7% |
| Precipitation | 855 mm yr^-1^ | 886 mm yr^-1^ | 884 mm yr^-1^ | 872 mm yr^-1^ | 3.6% | 3.4% | 2.0% |
|  | 866 mm yr^-1^ | 862 mm yr^-1^ | 861 mm yr^-1^ | 847 mm yr^-1^ | -0.4% | -0.5% | -2.2% |
|  | 856 mm yr^-1^ | 853 mm yr^-1^ | 847 mm yr^-1^ | 782 mm yr^-1^ | -0.4% | -1.1% | -8.6% |
| Snow storage | 11.1 mm | 8.7 mm | 7.5 mm | 7.1 mm | -21.4% | -32.8% | -36.0% |
|  | 11.3 mm | 7.5 mm | 5.3 mm | 4.8 mm | -33.7% | -52.5% | -57.5% |
|  | 11.3 mm | 7.4 mm | 4.7 mm | 1.9 mm | -34.5% | -58.0% | -83.1% |

**Table S8.** Discharge and sediment parameters for RCPs 2.6 (green), 4.5 (blue), and 8.5 (red).

|  | Changes compared to reference period | | |
| --- | --- | --- | --- |
| Devoll at Kokel | 2011-2040 | 2041-2070 | 2071-2100 |
| Discharge | 5.6% | 3.2% | -0.3% |
|  | -4.4% | -5.0% | -10.7% |
|  | -5.7% | -7.6% | -23.1% |
| Suspended Sediment Yield | 8.4% | 9.4% | 7.6% |
|  | 0.4% | 3.6% | 1.7% |
|  | 5.2% | 5.5% | -2.6% |
| Suspended Sediment Concentration | 3% | 6% | 8% |
|  | 5% | 9% | 14% |
|  | 12% | 14% | 27% |
| Devoll tributary | 2011-2040 | 2041-2070 | 2071-2100 |
| Discharge | 6.1% | 3.8% | -0.8% |
|  | -2.6% | -4.1% | -8.9% |
|  | -4.8% | -5.8% | -21.6% |
| Susp. Sed. Yield | 8.2% | 9.1% | 6.6% |
|  | 0.6% | 3.0% | 1.5% |
|  | 5.1% | 5.3% | -3.1% |
| Susp. Sed. Concentration | 2% | 5% | 7% |
|  | 3% | 7% | 11% |
|  | 10% | 12% | 24% |

**Table S9**. Discharge and sediment parameters for SSP1 (green), SSP2 (dark blue), SSP4 (light blue), and SSP5 (red).

|  | Changes compared to reference period | | |
| --- | --- | --- | --- |
| Devoll at Kokel | 2011-2040 | 2041-2070 | 2071-2100 |
| Suspended Sediment Yield | 7.9% | -2.5% | -7.5% |
|  | 3.4% | 21.4% | 41.0% |
|  | 1.8% | 12.1% | 18.9% |
|  | 5.3% | 12.7% | 8.1% |
| Suspended Sediment Concentration | 2.2% | -5.6% | -7.2% |
|  | 8.1% | 27.8% | 58.0% |
|  | 6.5% | 18.0% | 33.2% |
|  | 11.6% | 21.9% | 40.6% |
| Devoll tributary | 2011-2040 | 2041-2070 | 2071-2100 |
| Suspended Sediment Yield | 7.2% | -5.1% | -10.7% |
|  | 3.8% | 23.3% | 46.7% |
|  | 1.8% | 12.0% | 20.1% |
|  | 5.0% | 12.9% | 8.0% |
| Suspended Sediment Concentration | 1.1% | -8.5% | -10.0% |
|  | 6.6% | 28.6% | 61.0% |
|  | 4.6% | 16.8% | 31.9% |
|  | 10.2% | 19.9% | 37.7% |

# References

1. Popke, D., Stevens, B. & Voigt, A. Climate and climate change in a radiative-convective equilibrium version of ECHAM6. *Journal of Advances in Modeling Earth Systems* **5**, 1–14 (2013).

2. Hazeleger, W. *et al.* EC-Earth: A Seamless Earth-System Prediction Approach in Action. *Bulletin of the American Meteorological Society* **91**, 1357–1364 (2010).

3. Collins, W. J. *et al.* Development and evaluation of an Earth-System model – HadGEM2. *Geoscientific Model Development* **4**, 1051–1075 (2011).

4. Kjellström, E. *et al.* Production and use of regional climate model projections – A Swedish perspective on building climate services. *Climate Services* **2–3**, 15–29 (2016).

5. van Meijgaard, E., van Ulft, L., Lenderink, G., de Roode, S. & Timmermans, R. *Refinement and application of a regional atmospheric model for climate scenario calculations of Western Europe*. https://library.wur.nl/WebQuery/wurpubs/fulltext/312258.

6. van Vuuren, D. P. *et al.* The representative concentration pathways: an overview. *Climatic Change* **109**, 5 (2011).

7. Riahi, K. *et al.* The Shared Socioeconomic Pathways and their energy, land use, and greenhouse gas emissions implications: An overview. *Glob. Environ. Change* **42**, 153–168 (2017).

8. Diodato, N. & Bellocchi, G. Estimating monthly (R)USLE climate input in a Mediterranean region using limited data. *J. Hydrol.* **345**, 224–236 (2007).

9. Mouris, K., Schwindt, S., Haun, S., Morales Oreamuno, M. F. & Wieprecht, S. Introducing seasonal snow memory into the RUSLE. *J Soils Sediments* (2022) doi:10.1007/s11368-022-03192-1.

10. Wischmeier, W. H. & Smith, D. D. *Predicting Rainfall Erosion Losses: A Guide to Conservation Planning*. (Department of Agriculture, Science and Education Administration, 1978).

11. Copernicus Land Monitoring Service. Corine Land Cover 2018 Version 2020_20u1. *European Environment Agency (EEA)* (2018) doi:https://land.copernicus.eu/pan-european/corine-land-cover/clc2018.

12. Chen, M. *et al.* Global land use for 2015–2100 at 0.05° resolution under diverse socioeconomic and climate scenarios. *Sci Data* **7**, 320 (2020).

13. Zhang, H. *et al.* An improved method for calculating slope length (λ) and the LS parameters of the Revised Universal Soil Loss Equation for large watersheds. *Geoderma* **308**, 36–45 (2017).

14. Panagos, P., Meusburger, K., Ballabio, C., Borrelli, P. & Alewell, C. Soil erodibility in Europe: A high-resolution dataset based on LUCAS. *Sci. Total Environ.* **479–480**, 189–200 (2014).

15. Hiederer, R. *Mapping soil properties for Europe: spatial representation of soil database attributes.* (European Commission. Joint Research Centre. Institute for Environment and Sustainability., 2013).

16. Fischer, G. *et al.* The harmonized world soil database v 1.2. *IIASA, Laxenburg, Austria and FAO, Rome, Italy* (2008) doi:https://www.fao.org/soils-portal/data-hub/soil-maps-and-databases/harmonized-world-soil-database-v12/en/.

17. Griffin, M. L., Beasley, D. B., Fletcher, J. J. & Foster, G. R. Estimating soil loss on topographically non-uniform field and farm units. *J. Soil Water Conserv.* **43**, 326–331 (1988).

18. Haan, C. T., Barfield, B. J. & Hayes, J. C. *Design Hydrology and Sedimentology for Small Catchments*. (Elsevier Science, 1994).

19. *Handbook of Erosion Modelling: Morgan/Handbook of Erosion Modelling*. (John Wiley & Sons, Ltd, 2010). doi:10.1002/9781444328455.

20. Panagos, P. *et al.* Towards estimates of future rainfall erosivity in Europe based on REDES and WorldClim datasets. *Journal of Hydrology* **548**, 251–262 (2017).

21. European Environment Agency. *Europe’s changing climate hazards - Wet and dry*. (Publications Office, 2021).

22. Ferro, V. & Porto, P. Sediment Delivery Distributed (SEDD) Model. *J. Hydrol. Eng.* **5**, 411–422 (2000).

23. Jain, M. K. & Kothyari, U. C. Estimation of soil erosion and sediment yield using GIS. *Hydrol. Sci. J.* **45**, 771–786 (2000).

24. Olsen, N. R. B. *A Three-Dimensional Numerical Model For Simulation Of Sediment Movements In Water Intakes With Multiblock Option. User’s Manual*. (2018).

25. Van Rijn, L. C. Sediment Transport, Part I: Bed Load Transport. *Journal of Hydraulic Engineering* **110**, 1431–1456 (1984).

26. Mouris, K., Morales Oreamuno, M. F. & Surac, J. BC_calc_SSIIM. (2023).

27. Ahrens, J. P. Simple Equations to Calculate Fall Velocity and Sediment Scale Parameter. *Journal of Waterway, Port, Coastal, and Ocean Engineering* **129**, 146–150 (2003).
